# National age-specific mortality trends for cervical and breast cancers in urban–rural areas of China from 2009 to 2021: a population-based analysis

**DOI:** 10.1186/s40779-024-00561-4

**Published:** 2024-08-13

**Authors:** Meng-Long Li, Jin-Lei Qi, Ya-Qi Ma, Wen Shu, Hui-Di Xiao, Li-Jun Wang, Peng Yin, Hao-Yan Guo, Sten H. Vermund, Mai-Geng Zhou, Yi-Fei Hu

**Affiliations:** 1 Department of Child, Adolescent Health and Maternal Care, School of Public Health, Capital Medical University, Beijing, 100069 China; 2 National Center for Chronic and Noncommunicable Disease Control and Prevention, Chinese Center for Disease Control and Prevention (China CDC), Beijing, 100050 China; 3 Department of Pathology, the First Medical Center, Chinese PLA General Hospital, Beijing, 100853 China; 4 Yale School of Public Health, Yale University, New Haven, CT 06510-3201 USA; 5 UNESCO Chair on Global Health and Education, Peking University, Beijing, 100083 China; 6 Beijing Key Laboratory of Environmental Toxicology, Capital Medical University, Beijing, 100069 China

**Keywords:** Cervical cancer, Breast cancer, Age-specific mortality, Trend, Urban–rural difference, Joinpoint model, China

## Abstract

**Background:**

Cervical and breast cancers are among the top 4 leading causes of cancer-related mortality in women. This study aimed to examine age-specific temporal trends in mortality for cervical and breast cancers in urban and rural areas of China from 2009 to 2021.

**Methods:**

Age-specific mortality data for cervical and breast cancers among Chinese women aged 20–84 years were obtained from China’s National Disease Surveillance Points system spanning the years 2009 to 2021. Negative binomial regression models were utilized to assess urban–rural differences in mortality rate ratios, while Joinpoint models with estimated average annual percent changes (AAPC) and slopes were employed to compare temporal trends and the acceleration of mortality rates within different age groups.

**Results:**

From 2009 to 2021, there was a relative increase in age-specific mortality associated with the two cancers observed in rural areas compared with urban areas. A rising trend in the screening age of 35–64 [AAPC: 4.0%, 95% confidence interval (CI) 0.5–7.6%, *P* = 0.026] for cervical cancer was noted in rural areas, while a stable trend (AAPC: − 0.7%, 95% CI − 5.8 to 4.6%, *P* = 0.78) was observed in urban areas. As for breast cancer, a stable trend (AAPC: 0.3%, 95% CI − 0.3 to 0.9%, *P* = 0.28) was observed in rural areas compared to a decreasing trend (AAPC: − 2.7%, 95% CI − 4.6 to − 0.7%, *P* = 0.007) in urban areas. Urban–rural differences in mortality rates increased over time for cervical cancer but decreased for breast cancer. Mortality trends for both cervical and breast cancers showed an increase with age across 4 segments, with the most significant surge in mortality observed among the 35–54 age group across urban and rural areas, periods, and regions in China.

**Conclusions:**

Special attention should be given to women aged 35–54 years due to mortality trends and rural–urban disparities. Focusing on vulnerable age groups and addressing rural–urban differences in the delivery of cancer control programs can enhance resource efficiency and promote health equity.

**Supplementary Information:**

The online version contains supplementary material available at 10.1186/s40779-024-00561-4.

## Background

Cervical and breast cancers, often referred to as the “two cancers”, are two of the 4 primary causes of cancer-related deaths among women [1]. According to the Global Cancer Observatory (GLOBOCAN), over one million women globally died from these two diseases in 2020 [2, 3] and 2022 [1]. In China, breast cancer had the highest incidence rate [age-standardized incidence rate (ASIR) ranked 1st] [4], while also ranking as the 5th leading cause of cancer-related deaths among women in 2020 [5]. Cervical cancer ranked 6th in ASIR [4] and was the 6th leading cause of cancer-related deaths among women in China in 2020 [5]. The global progress made in reducing the incidence and mortality rates of cervical and breast cancers underscores the critical need to increase human papillomavirus (HPV) vaccination coverage, implement comprehensive cancer screening programs, and ensure prompt treatment [6, 7]. In China, a nationwide screening initiative for cervical and breast cancers, known as the “two cancers” screening program (referred to as the Program), has been in operation since 2009, alongside with approval of the HPV vaccine in 2016 [8]. The Program was progressively expanded to cover women aged 35–64 years in both urban and rural areas in China from 2009 to 2022 [9–11]. However, due to budget constraints and a vast number of eligible women in rural areas, only a fraction of the targeted population in designated counties has been reached [12, 13]. Hence, achieving the ambitious goal of eliminating these two cancers appears challenging, given the present level of health investments, population coverage, and policy frameworks.

The incidence and mortality rates of the two cancers are directly correlated with age [7, 14–18]. Globally, the average age at diagnosis for cervical cancer is 53 years [14], while in China, it ranges from 48 to 54 years [19]. The median age at breast cancer diagnosis in China is 48 to 50 years, notably younger than in the United States where it is 64 years old [18]. The global average age of death from cervical cancer is 59 years [14]. However, these averages conceal trends, particularly within age groups experiencing increases in cervical or breast cancer mortality. Understanding temporal trends in age-specific mortality, especially for women aged 35–64 years covered by the Program, and cancer-specific mortality, and delineating the accelerating rates of mortality rates by age for these two cancers can help establish public health priorities in China.

Despite the implementation of the Program, significant disparities in mortality rates between urban and rural areas persist for both cervical and breast cancers [20], primarily due to limited access to screening and underlying social determinants of health. Biological and social determinants such as HPV infection, other sexually transmittable infections (STIs), smoking, oral hormonal contraception, early sexual debut, multiple sexual partners, and inadequate screening or vaccination are associated with cervical cancer [2, 6, 14]. For breast cancer, risk factors include early age at menarche, advanced age at first childbirth, later menopausal age, fewer children, less breastfeeding, menopausal hormone therapy, oral contraceptives, excess body weight, and physical inactivity, which are typically more prevalent in urban areas [2, 7]. Moreover, women in rural areas face higher risks due to factors like immunosuppression, genetic predilections, and poverty [2, 6, 7].

The objective of this study is to identify age-specific mortality temporal trends in both urban and rural areas, as well as to evaluate the acceleration in mortality rates by age for cervical and breast cancers in China from 2009 to 2021. By identifying trends and urban–rural differences in age-specific mortality rates for the two types of cancers, this research may contribute to efforts aimed at achieving health equity.

## Methods

### Data source

Mortality data for women, including the number of deaths due to cervical cancer [International Classification of Diseases, 10th revision (ICD-10): C53] and breast cancer (ICD-10: C50) from 2009 to 2021, categorized by age, residence, and region, were obtained from China’s National Diseases Surveillance Points (DSP) system. This system is established and managed by the Chinese Center for Disease Control and Prevention (China CDC). After a significant upgrade in 2013, the DSP system expanded its surveillance points to 605, covering urban (208 points) and rural (397 points) areas across all 31 provinces/autonomous regions/municipalities (hereafter termed provinces) of the Chinese mainland. This coverage encompasses nearly one-quarter of the population and is described in detail elsewhere [21–23] and in Additional file 1. National Diseases Surveillance Points (DSP) system. In China, the eastern region includes 11 provinces (Beijing, Shanghai, Tianjin, Guangdong, Shandong, Jiangsu, Zhejiang, Fujian, Hainan, Hebei, and Liaoning), the central region includes 8 provinces (Henan, Jilin, Anhui, Heilongjiang, Hunan, Hubei, Jiangxi, and Shanxi), the 12 western provinces include Guangxi, Shaanxi, Gansu, Qinghai, Sichuan, Yunnan, Guizhou, Chongqing, Inner Mongolia, Ningxia, Tibet (Xizang), and Xinjiang. Eastern China is the most economically developed region, while Western China is the least developed.

Deaths are reported from all locations, including hospitals, homes, and other settings. The causes of death are determined using a standardized protocol by trained personnel based in local hospitals or CDC branches. Previous studies have shown the DSP system’s robust national and regional representativeness, consistently providing reliable reporting of cancer-related deaths [24–26]. To ensure data validity and consistency, annual training on a standardized protocol is conducted, which includes random checks for disease classification accuracy and duplications. Retrospective surveys are also carried out to assess underreporting and ensure the completeness and accuracy of disease coding. Underreporting surveys are conducted every three years to evaluate the completeness of the DSP system and correct any potential underreporting bias [21]. Age-specific population data for each DSP were obtained from the National Bureau of Statistics of China (http://data.stats.gov.cn) to calculate mortality rates. The variables in this study included disease type (cervical cancer or breast cancer), mortality rates, age, residence (urban or rural areas), and region (Eastern, Central, or Western China).

Considering the diagnosis and incidence characteristics of the two cancers, analysis was restricted to women aged 20–84 years. Exclusion criteria encompassed women aged 0–19 years and those aged 85 years and above. Mortality among women aged 0–19 years is exceedingly rare, while individuals aged 85 years and older often present with comorbidities and competing risks, rendering mortality analysis complex. Age data were categorized into 5-year intervals in the system, and 3 sub-age groups were generated to assess mortality trends: those covered by the Program (35–64 years), and younger (20–34 years) or older (65–84 years) population. The study utilized de-identified information from approximately 130 million women obtained from a publicly available database. Given that the data were anonymized and no clinical trials, subject recruitment, or intervention were involved in this study, it was exempted from institutional review board (IRB) review by both Capital Medical University and the China CDC.

### Statistical analysis

The reported mortality rates were estimated by age, residence, and region, adjusting for underreporting rates in each corresponding year as per the following formula: mortality rate = crude mortality rate/(1 − underreporting rate). Pooled average annual mortality rates were calculated with exact Poisson 95% confidence intervals (CIs) by period and region to conduct stratified analyses and generate sunburst charts of provincial rankings. Log-linear Joinpoint model was used to determine temporal trends in overall and age-specific mortality rates for cervical cancer and breast cancer based on the formula: $\text{ln}\left(y\right)= \beta x+\varepsilon$, where $y$ is the mortality rate, $x$ is the calendar year, $\beta$ is the slope coefficient and $\varepsilon$ is the error term, with no more than two Joinpoints as per guidelines [27]. Annual percent changes (APCs) were estimated as $[\text{exp}\left(\beta \right)-1]\times 100$, and average annual percent changes (AAPCs) were estimated as $[\text{exp}\left(\frac{\sum {w}_{i}{\beta }_{i}}{\sum {w}_{i}}\right)-1]\times 100$, where $i$ is the segments in the desired range of calendar years, ${w}_{i}$ is the length of each segment in the range of years and ${\beta }_{i}$ is the slope coefficient for the $i$ th segment, and 95% CI can be obtained from the Joinpoint models [27].

Negative binomial regression models were used to assess the mortality rate ratios and 95% CIs between urban and rural areas (with rural as the reference) based on the formula: $\text{log}\left(\mu /n\right)={\beta }_{0}+{\beta }_{1}{R}_{urban}+{\beta }_{2}{R}_{rural}$, where $\mu$ is the total deaths attributed to cervical cancer or breast cancer in a specific age group, region, or period, $n$ is the corresponding population and ${R}_{urban}$ or ${R}_{rural}$ are 0–1 coded dummy variables [28]. Linear Joinpoint models were used to estimate slopes of the mortality rate trends by age based on the formula: $y= \beta x+\varepsilon$, where $y$ is the mortality rate, $x$ represents the age group, $\beta$ is the slope coefficient and $\varepsilon$ is the error term, with three Joinpoints [27]. Then the mortality accelerating age ranges of the two cancers were identified and modeled mortality rates from the Joinpoint models were layered to generate the figures. For comparing the patterns of mortality acceleration with age for cervical and breast cancers to all cancers, the age-specific mortality rates of all cancers among women in urban and rural areas in 2017 were extracted and modeled as the reference [29].

The *Z* test assessed the significance of the APC, AAPC, and slope from zero, the Chi-square test assessed the significance of the rate ratios between urban and rural areas, and a two-tailed *P* of 0.05 was deemed statistically significant. An increasing trend is defined as when the APC or AAPC and its lower 95% CI are both greater than zero. A decreasing trend is defined as when the APC or AAPC and its upper 95% CI are both less than zero. If the 95% CI contain zero, it is defined as a stable trend. Data management, analysis, and figure processing were performed using Excel (version Microsoft 365, Microsoft Corp., Washington, DC, USA), Statistical Analysis System® (version 9.4, SAS Institute Inc., Cary, NC, USA), Joinpoint Regression Program (version 4.9, the National Cancer Institute, Rockville, MD, USA), and R software (version 3.4.1, R Foundation, Vienna, Austria).

## Results

### Overall trends in mortality rates for cervical cancer and breast cancer

Among women aged 20–84 years in China, the pooled mortality rates from 2009 to 2021 were 6.29 per 100,000 for cervical cancer and 9.76 per 100,000 for breast cancer, adjusted for underreporting rates in different years (Additional file 1: Table S1). Temporal trends showed that the years 2013 and 2017 were significant Joinpoints for stratified analysis by period. The APCs for cervical cancer and breast cancer were at 3.1% (95% CI − 0.1 to 6.5, *P* = 0.06) and − 2.6% (95% CI − 4.4 to − 0.8, *P* = 0.015) during 2009–2013, 12.0% (95% CI 6.5–17.8, *P* = 0.002) and 2.8% (95% CI − 0.2 to 5.9, *P* = 0.06) during 2013–2017, and − 5.5% (95% CI − 8.4 to − 2.4, *P* = 0.006) and − 0.2% (95% CI − 2.1 to 1.6, *P* = 0.75) during 2017–2021, respectively (Additional file 1: Table S2).

### Temporal trends in age-specific mortality rates by residence

Between the years of 2009–2021, a notable increase was observed in age-specific mortality rates for cervical cancer among women aged 35 to 64 residing in rural regions (AAPC: 4.0%, 95% CI 0.5–7.6, *P* = 0.026), whereas these rates remained steady within urban locales (AAPC: − 0.7%, 95% CI − 5.8 to 4.6, *P* = 0.78). In 2021, the mortality rate due to cervical cancer within this age group stood at 5.06 per 100,000 in urban areas and 7.09 per 100,000 in rural areas. For breast cancer in the same age group, stability was noted across rural areas (AAPC: 0.3%, 95% CI − 0.3 to 0.9, *P* = 0.28), while a decline was observed within urban areas (AAPC: − 2.7%, 95% CI − 4.6 to − 0.7, *P* = 0.007). In 2021, the mortality rate for breast cancer among women aged 35–64 was 10.08 per 100,000 in urban areas and 10.91 per 100,000 in rural areas (Table 1). Temporal trends in age-specific mortality rates for cervical and breast cancer from 2009 to 2021 are depicted in Additional file 1: Fig. S1. There is a notable increasing trend in mortality rate for cervical cancer among women aged 55–59 years in both urban areas (AAPC: 2.9%, 95% CI 0.7–5.2, *P* = 0.015) and rural areas (AAPC: 4.5%, 95% CI 2.9–6.0, *P* < 0. 001) (Additional file 1: Table S3).

**Table 1:** Temporal trends in age-specific mortality rates for cervical cancer and breast cancer in China, 2009–2021

Age group (years)	Mortality rate (1/100,000, 95% CI)								AAPC [%(95% CI)]		Pooled mortality rate (1/100,000, 95% CI)		
	2009		2013		2017		2021		2009–2021		2009–2021		
	Urban	Rural	Urban	Rural	Urban	Rural	Urban	Rural	Urban	Rural	Urban	Rural	Ratio
Cervical cancer													
20–34	0.73 (0.46–1.09)	0.38 (0.23–0.58)	0.35 (0.27–0.45)	0.38 (0.31–0.48)	0.38 (0.29–0.48)	0.56 (0.47–0.67)	0.29 (0.22–0.39)	0.45 (0.35–0.56)	− 4.5 (− 7.3 to − 1.6)*	0.4 (− 3.1 to 4.1)	0.43 (0.40–0.46)	0.56 (0.52–0.59)	0.78 (0.71–0.85)*
35–64	5.59 (5.03–6.19)	4.66 (4.25–5.11)	4.99 (4.73–5.26)	5.12 (4.91–5.34)	6.82 (6.53–7.12)	8.77 (8.49–9.05)	5.06 (4.83–5.31)	7.09 (6.84–7.36)	− 0.7 (− 5.8 to 4.6)	4.0 (0.5–7.6)*	6.12 (6.03–6.21)	7.37 (7.29–7.45)	0.83 (0.82–0.85)*
65–84	10.98 (9.32–12.86)	13.58 (11.97–15.35)	9.98 (9.17–10.84)	12.05 (11.33–12.79)	15.25 (14.34–16.21)	19.98 (19.14–20.85)	11.52 (10.84–12.22)	17.97 (17.26–18.71)	0.5 (− 5.5 to 6.8)	2.4 (− 0.8 to 5.7)	13.38 (13.11–13.66)	18.23 (17.97–18.49)	0.73 (0.72–0.75)*
Breast cancer													
20–34	1.16 (0.81–1.59)	0.73 (0.52–0.99)	0.70 (0.57–0.84)	0.79 (0.68–0.92)	0.83 (0.70–0.97)	0.94 (0.81–1.07)	0.79 (0.66–0.94)	1.08 (0.93–1.25)	− 3.1 (− 7.1 to 1.1)	2.5 (1.3–3.8)*	0.74 (0.70–0.78)	0.90 (0.86–0.94)	0.82 (0.77–0.88)*
35–64	13.73 (12.86–14.66)	10.81 (10.17–11.48)	12.01 (11.61–12.42)	9.91 (9.61–10.21)	13.07 (12.66–13.49)	11.51 (11.19–11.84)	10.08 (9.75–10.43)	10.91 (10.58–11.23)	− 2.7 (− 4.6 to − 0.7)*	0.3 (− 0.3 to 0.9)	12.17 (12.05–12.30)	10.91 (10.81–11.01)	1.12 (1.10–1.13)*
65–84	32.57 (29.66–35.69)	17.13 (15.31–19.10)	27.78 (26.42–29.19)	15.25 (14.44–16.09)	29.25 (27.97–30.56)	18.11 (17.31–18.93)	27.06 (26.02–28.13)	18.02 (17.31–18.76)	− 1.6 (− 2.9 to − 0.3)*	1.2 (0.3–2.1)*	28.59 (28.19–28.99)	17.42 (17.17–17.67)	1.64 (1.61–1.67)*

^*^P < 0.05, the *Z* test was used to identify the significance of the AAPC obtained from the log-linear Joinpoint model from zero, the Chi-square test was used to identify the significance of the mortality rate ratios between urban and rural (reference) areas obtained from the negative binomial regression model from one. *CI* confidence interval, *AAPC* average annual percent change

In the 20–34-year age group, the cervical cancer mortality rate decreased in urban areas (AAPC: − 4.5%, 95% CI − 7.3 to − 1.6, *P* = 0.006) and remained stable in rural areas (AAPC: 0.4%, 95% CI − 3.1 to 4.1, *P* = 0.79), while the breast cancer mortality rate remained stable in urban areas (AAPC: − 3.1%, 95% CI − 7.1 to 1.1, *P* = 0.13) and increased in rural areas (AAPC: 2.5%, 95% CI 1.3–3.8, *P* = 0.001). In 2021, the mortality rates for cervical cancer and breast cancer among women in this age group were 0.29 and 0.79 per 100,000 in urban areas, and 0.45 and 1.08 per 100,000 in rural areas, respectively (Table 1).

In the 65–84-year-old age group, cervical cancer mortality rates remained stable in both urban areas (AAPC: 0.5%, 95% CI − 5.5 to 6.8, *P* = 0.88) and rural areas (AAPC: 2.4%, 95% CI − 0.8 to 5.7, *P* = 0.14). However, an increasing trend for breast cancer was observed in rural areas (AAPC: 1.2%, 95% CI 0.3–2.1, *P* = 0.012), while a decreasing trend was seen in urban areas (AAPC: − 1.6%, 95% CI − 2.9 to − 0.3, *P* = 0.013). In 2021, the mortality rates for cervical cancer and breast cancer among women aged 65–84 years were 11.52 and 27.06 per 100,000 in urban areas, and 17.97 and 18.02 per 100,000 in rural areas, respectively (Table 1). There is a notable increasing trend in the mortality rate for cervical cancer among women aged 70–74 years in urban areas (AAPC: 4.7%, 95% CI 0.3–9.2, *P* = 0.035) and women aged 75–79 years in rural areas (AAPC: 6.1%, 95% CI 3.7–8.6, *P* < 0. 001) (Additional file 1: Table S3). As for breast cancer, there is a significant upward trend in mortality rate among women aged 70–74 years (AAPC: 2.2%, 95% CI 0.6–3.9, *P* = 0.012) and 75–79 years (AAPC: 2.1%, 95% CI 0.2–4.0, *P* = 0.036) in rural areas (Additional file 1: Table S3).

### Differences in age-specific mortality rates between urban and rural areas

There were relatively minor variations in mortality rates between urban and rural areas (with rural as the reference) among individuals aged 35–64 years (rate ratios: 0.83 for cervical cancer and 1.12 for breast cancer), compared to more pronounced differences observed within younger (rate ratios: 0.78 for cervical cancer and 0.82 for breast cancer) and older groups (rate ratios: 0.73 for cervical cancer and 1.64 for breast cancer) (Table 1). For cervical cancer, the disparity in mortality rate between urban and rural (reference) areas was statistically significant across all age groups, with rate ratios ranging from 0.55 to 0.93. This difference peaked in the group aged 20–24 (rate ratio: 0.55), reached its lowest point in those aged 40–44 (rate ratio: 0.93), and then changed with age to a rate ratio of 0.67 in the 70–74-year group, and 0.82 in the 80–84-year group. For breast cancer, nearly all age groups (rate ratio: 0.72 to 2.29) exhibited an increasing trend with age, except for those aged 20–24 (rate ratio: 0.74), and 40–44 (rate ratio: 1.01), which showed the lowest differences and did not demonstrate significant variation over the period from 2009 to 2021 (Additional file 1: Table S3).

After stratification by period and region, significant disparities were found in cervical cancer mortality between urban and rural (reference) areas among women aged 20–84 years, with rate ratios ranging from 0.69 to 0.92, indicating higher rates observed in rural areas compared to urban areas. Conversely, lower rates were noted for breast cancer mortality in rural areas compared to urban areas, with rate ratios ranging from 1.11 to 1.32. Over time, the mortality disparities for cervical cancer between urban and rural areas changed from a rate ratio of 0.92 in 2009–2013, to 0.78 in 2013–2017 and further to 0.69 in 2017–2021. Conversely, for breast cancer, the disparities changed from a rate ratio of 1.32 in 2009–2013 to 1.23 in 2013–2017, and then to 1.11 in 2017–2021. Regionally, the disparity in cervical cancer mortality between urban and rural areas was most pronounced in Eastern China (rate ratio: 0.71), followed by Central China (rate ratio: 0.80), and Western China (rate ratio: 0.84). For breast cancer, the most significant disparities between urban and rural areas were observed in Central China (rate ratio: 1.27), followed by Western China (rate ratio: 1.15), and then Eastern China (rate ratio: 1.14) **(**Additional file 1: Table S4). In the 35–64-year age group, mortality disparities between urban and rural areas for cervical cancer changed from a rate ratio of 1.00 in 2009–2013, to 0.86 in 2013–2017, and further to 0.77 in 2017–2021. For breast cancer, mortality disparities changed from a rate ratio of 1.20 in 2009–2013, to 1.18 in 2013–2017, and then to 1.05 in 2017–2021. Regionally, the greatest differences in mortality between urban and rural areas were found in Western China (rate ratio: 0.83) for cervical cancer and Central China for breast cancer (rate ratio: 1.20) (Additional file 1: Table S4). Detailed age-specific mortality differences are presented in Additional file 1: Fig. S2, Tables S5 and S6. The changes by period and region align with the overall findings, indicating that cervical cancer mortality tends to be higher in rural areas than in urban areas, while breast cancer mortality is higher in urban areas than that in rural areas.

**Fig. 1:**
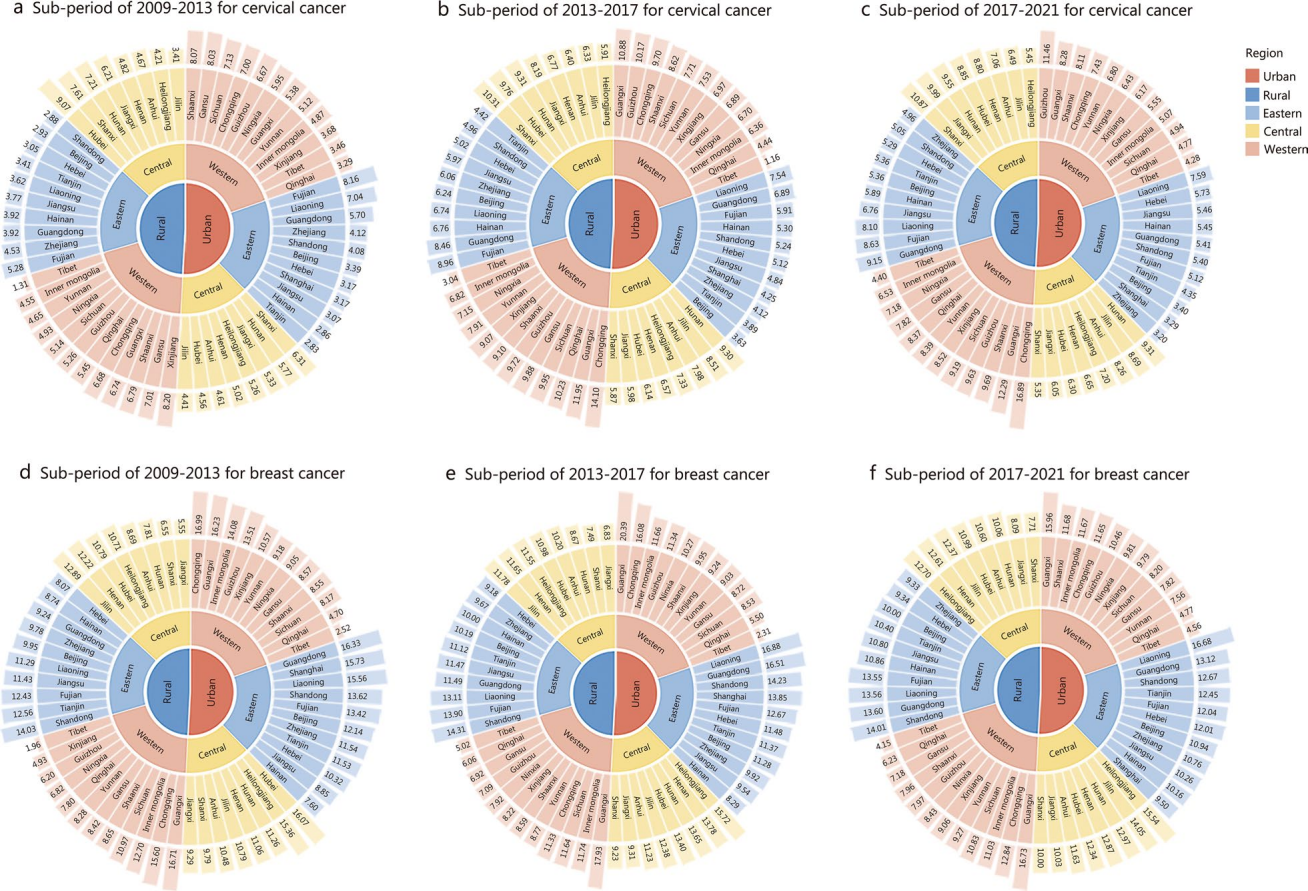
Province ranking in mortality rates per 100,000 women for cervical cancer and breast cancer among women aged 35–64 years in urban and rural areas, by period and region, in China, 2009–2021. In the inner central ring, the rural provinces number is fewer than the urban provinces, since Shanghai has no rural sites

Considerable heterogeneity in the mortality rates of cervical and breast cancer was found between urban and rural areas among women aged 35–64 years, with rate ratios of 0.83 (95% CI 0.82–0.85) for cervical cancer and 1.12 (95% CI 1.10–1.13) for breast cancer (Table 1). Stratified by period and region, in urban areas, the highest cervical cancer mortality rates in Eastern, Central and Western China were in Fujian (8.16 per 100,000), Shanxi (6.31 per 100,000), and Shaanxi (8.07 per 100,000) in 2009–2013. In 2013–2017, the highest rates were in Liaoning (7.54 per 100,000), Hunan (9.30 per 100,000), and Guangxi (10.88 per 100,000). In 2017–2021, the highest rates were in Liaoning (7.59 per 100,000), Hunan (9.31 per 100,000), and Guizhou (11.46 per 100,000). In rural areas, the highest rates in 2009–2013 were in Fujian (5.28 per 100,000), Hubei (9.07 per 100,000), and Xinjiang (8.20 per 100,000). In 2013–2017, the highest rates were in Fujian (8.96 per 100,000), Shanxi (10.31 per 100,000), and Chongqing (14.10 per 100,000). In 2017–2021, the highest rates were in Guangdong (9.15 per 100,000), Shanxi (10.87 per 100,000), and Chongqing (16.89 per 100,000; Fig. 1a–c). In urban areas, the highest breast cancer mortality rates in Eastern, Central, and Western China in 2009–2013 were in Guangdong (16.33 per 100,000), Hubei (16.07 per 100,000), and Chongqing (16.99 per 100,000). In 2013–2017, the highest rates were in Liaoning (16.88 per 100,000), Heilongjiang (15.72 per 100,000), and Guangxi (20.39 per 100,000). In 2017–2021 the highest rates were in Liaoning (16.68 per 100,000), Heilongjiang (15.54 per 100,000), and Guangxi (15.96 per 100,000). In rural areas, the highest breast cancer rates in 2009–2013 occurred in Shandong (14.03 per 100,000), Jilin (12.89 per 100,000), and Guangxi (16.71 per 100,000). In 2013–2017, the highest rates were in Shandong (14.31 per 100,000), Jilin (11.78 per 100,000), and Guangxi (17.93 per 100,000). In 2017–2021, the highest rates were in Shandong (14.01 per 100,000), Heilongjiang (12.70 per 100,000), and Guangxi (16.73 per 100,000; Fig. 1d–f).

The provincial mortality disparities between urban and rural areas for the two cancers among women aged 35–64 years changed over time (Table 2). In Guangdong, Eastern China, the mortality disparity of cervical cancer between urban and rural areas changed from 1.45 in 2009–2013, to 0.81 in 2013–2017, and further to 0.59 in 2017–2021. In Fujian, Eastern China, the mortality disparity of breast cancer between urban and rural areas changed from 1.08 in 2009–2013, to 0.91 in 2013–2017, and then to 0.89 in 2017–2021. In Shanxi, Central China, the mortality disparity of cervical cancer between urban and rural areas changed from 0.83 in 2009–2013, to 0.57 in 2013–2017, and further to 0.49 in 2017–2021. In Henan, Central China, the mortality disparity of breast cancer between urban and rural areas changed from 0.90 in 2009–2013, to 1.18 in 2013–2017, and then to 1.02 in 2017–2021. In Chongqing, Western China, the mortality disparity of cervical cancer between urban and rural areas changed from 1.05 in 2009–2013, to 0.69 in 2013–2017, and further to 0.44 in 2017–2021. In Sichuan, Western China, the mortality disparity of breast cancer between urban and rural areas changed from 0.75 in 2009–2013, to 0.73 in 2013–2017, and then to 0.76 in 2017–2021. Notable changes were observed in the urban–rural differences in mortality rate ratios in Tibet (from 2.64 to 0.97) for cervical cancer and in Xinjiang (from 2.14 to 1.08) for breast cancer (Table 2).

**Table 2:** Age-specific mortality rate ratios for cervical cancer and breast cancer among women aged 35–64 years between urban and rural areas, period-specific and province-specific in China, 2009–2021

Province	Cervical cancer rate ratio (95% CI)			Breast cancer rate ratio (95% CI)		
	2009–2013	2013–2017	2017–2021	2009–2013	2013–2017	2017–2021
Eastern						
Beijing	1.16 (0.50–2.70)	0.58 (0.44–0.77)^*^	0.63 (0.47–0.85)^*^	1.22 (0.77–1.93)	1.11 (0.90–1.36)	1.09 (0.89–1.34)
Tianjin	0.83 (0.53–1.29)	0.88 (0.65–1.20)	0.81 (0.60–1.09)	0.92 (0.73–1.15)	1.02 (0.84–1.24)	1.20 (0.97–1.47)
Guangdong	1.45 (1.18–1.80)^*^	0.81 (0.74–0.89)^*^	0.59 (0.54–0.65)^*^	1.77 (1.55–2.02)^*^	1.44 (1.34–1.54)^*^	0.97 (0.90–1.03)
Shandong	1.42 (1.16–1.73)^*^	1.06 (0.95–1.17)	1.07 (0.96–1.19)	0.97 (0.88–1.07)	0.99 (0.93–1.06)	0.90 (0.85–0.97)^*^
Jiangsu	0.82 (0.64–1.04)	0.81 (0.73–0.90)^*^	0.81 (0.73–0.89)^*^	0.77 (0.67–0.89)^*^	0.83 (0.77–0.90)^*^	0.95 (0.88–1.02)
Zhejiang	0.91 (0.68–1.22)	0.68 (0.54–0.86)^*^	0.65 (0.50–0.83)^*^	1.18 (0.98–1.41)	1.03 (0.88–1.20)	1.15 (1.00–1.33)
Fujian	1.54 (1.20–1.99)^*^	0.66 (0.57–0.76)^*^	0.59 (0.51–0.69)^*^	1.08 (0.90–1.30)	0.91 (0.82–1.01)	0.89 (0.80–0.99)^*^
Hainan	0.73 (0.40–1.34)	0.78 (0.57–1.08)	0.93 (0.67–1.28)	0.87 (0.59–1.28)	0.83 (0.64–1.08)	0.93 (0.74–1.19)
Hebei	1.04 (0.75–1.43)	1.02 (0.85–1.23)	1.08 (0.92–1.28)	1.28 (1.06–1.54)^*^	1.25 (1.10–1.42)^*^	1.29 (1.15–1.44)^*^
Liaoning	1.94 (1.54–2.45)^*^	1.12 (0.98–1.28)	0.94 (0.82–1.07)	1.38 (1.19–1.59)^*^	1.29 (1.18–1.41)^*^	1.23 (1.12–1.35)^*^
Central						
Henan	1.04 (0.84–1.29)	0.97 (0.86–1.09)	0.76 (0.68–0.84)^*^	0.90 (0.79–1.04)	1.18 (1.09–1.28)^*^	1.02 (0.94–1.11)
Jilin	1.29 (0.92–1.82)	1.34 (1.12–1.62)^*^	1.34 (1.12–1.61)^*^	0.84 (0.68–1.02)	1.05 (0.91–1.22)	1.14 (0.99–1.31)
Anhui	0.99 (0.78–1.25)	1.25 (1.11–1.40)^*^	1.17 (1.05–1.31)^*^	1.21 (1.03–1.42)^*^	1.10 (1.00–1.21)^*^	1.10 (1.00–1.21)
Heilongjiang	1.25 (0.97–1.61)	1.24 (1.08–1.42)^*^	1.32 (1.15–1.52)^*^	1.43 (1.23–1.68)^*^	1.36 (1.24–1.50)^*^	1.22 (1.11–1.35)^*^
Hunan	0.80 (0.65–0.98)^*^	1.00 (0.90–1.11)	0.97 (0.88–1.08)	1.44 (1.23–1.69)^*^	1.57 (1.43–1.73)^*^	1.29 (1.18–1.41)^*^
Hubei	0.50 (0.40–0.63)^*^	0.63 (0.55–0.72)^*^	0.71 (0.62–0.82)^*^	1.49 (1.29–1.72)^*^	1.22 (1.10–1.35)^*^	1.12 (1.01–1.25)^*^
Jiangxi	0.86 (0.65–1.13)	0.73 (0.62–0.86)^*^	0.61 (0.52–0.71)^*^	1.68 (1.30–2.15)^*^	1.36 (1.17–1.59)^*^	1.24 (1.08–1.43)^*^
Shanxi	0.83 (0.64–1.07)	0.57 (0.47–0.68)^*^	0.49 (0.41–0.59)^*^	1.50 (1.19–1.89)^*^	1.23 (1.05–1.45)^*^	1.30 (1.11–1.51)^*^
Western						
Guangxi	0.80 (0.59–1.07)	0.91 (0.80–1.04)	0.67 (0.59–0.78)^*^	0.97 (0.82–1.16)	1.14 (1.03–1.26)^*^	0.95 (0.86–1.06)
Shaanxi	1.19 (0.87–1.62)	0.95 (0.79–1.13)	0.84 (0.70–1.00)	0.99 (0.74–1.32)	1.16 (0.97–1.38)	1.47 (1.22–1.76)^*^
Gansu	1.15 (0.89–1.48)	0.70 (0.60–0.81)^*^	0.71 (0.60–0.84)^*^	1.02 (0.80–1.29)	1.26 (1.08–1.48)^*^	1.09 (0.93–1.28)
Qinghai	0.60 (0.30–1.21)	0.43 (0.29–0.64)^*^	0.57 (0.39–0.83)^*^	0.60 (0.34–1.07)	0.91 (0.61–1.34)	0.77 (0.52–1.14)
Sichuan	1.39 (1.14–1.69)^*^	0.77 (0.70–0.86)^*^	0.54 (0.47–0.61)^*^	0.75 (0.63–0.88)^*^	0.73 (0.66–0.81)^*^	0.76 (0.68–0.84)^*^
Yunnan	1.10 (0.77–1.57)	0.95 (0.79–1.14)	0.81 (0.67–0.98)^*^	1.11 (0.85–1.45)	1.03 (0.87–1.22)	0.82 (0.68–0.97)^*^
Guizhou	1.27 (0.93–1.73)	1.05 (0.90–1.21)	1.19 (1.05–1.35)^*^	2.18 (1.71–2.79)^*^	1.60 (1.38–1.86)^*^	1.31 (1.14–1.51)^*^
Chongqing	1.05 (0.81–1.36)	0.69 (0.60–0.78)^*^	0.44 (0.38–0.50)^*^	1.09 (0.92–1.29)	1.42 (1.24–1.62)^*^	0.91 (0.79–1.04)
Inner Mongolia	1.07 (0.76–1.52)	0.93 (0.77–1.13)	0.78 (0.63–0.96)^*^	1.11 (0.90–1.36)	0.99 (0.86–1.15)	1.06 (0.91–1.22)
Ningxia	1.21 (0.76–1.91)	0.94 (0.73–1.21)	0.90 (0.70–1.15)	1.33 (0.90–1.95)	1.30 (1.03–1.63)^*^	1.16 (0.94–1.44)
Tibet	2.64 (0.71–9.87)	0.38 (0.12–1.20)	0.97 (0.51–1.84)	1.28 (0.39–4.27)	0.46 (0.20–1.06)	1.10 (0.58–2.08)
Xinjiang	0.45 (0.30–0.67)^*^	0.77 (0.60–0.98)^*^	0.72 (0.57–0.92)^*^	2.14 (1.58–2.92)^*^	1.12 (0.90–1.40)	1.08 (0.88–1.32)

^*^P < 0.05, the Chi-square test was used to identify the significance of the mortality rate ratios between urban and rural (reference) areas obtained from the negative binomial regression model from one. Due to no rural sites in Shanghai, mortality rate ratios cannot be calculated. *CI* confidence interval

### Mortality surge age ranges in urban and rural China

**Fig. 2:**
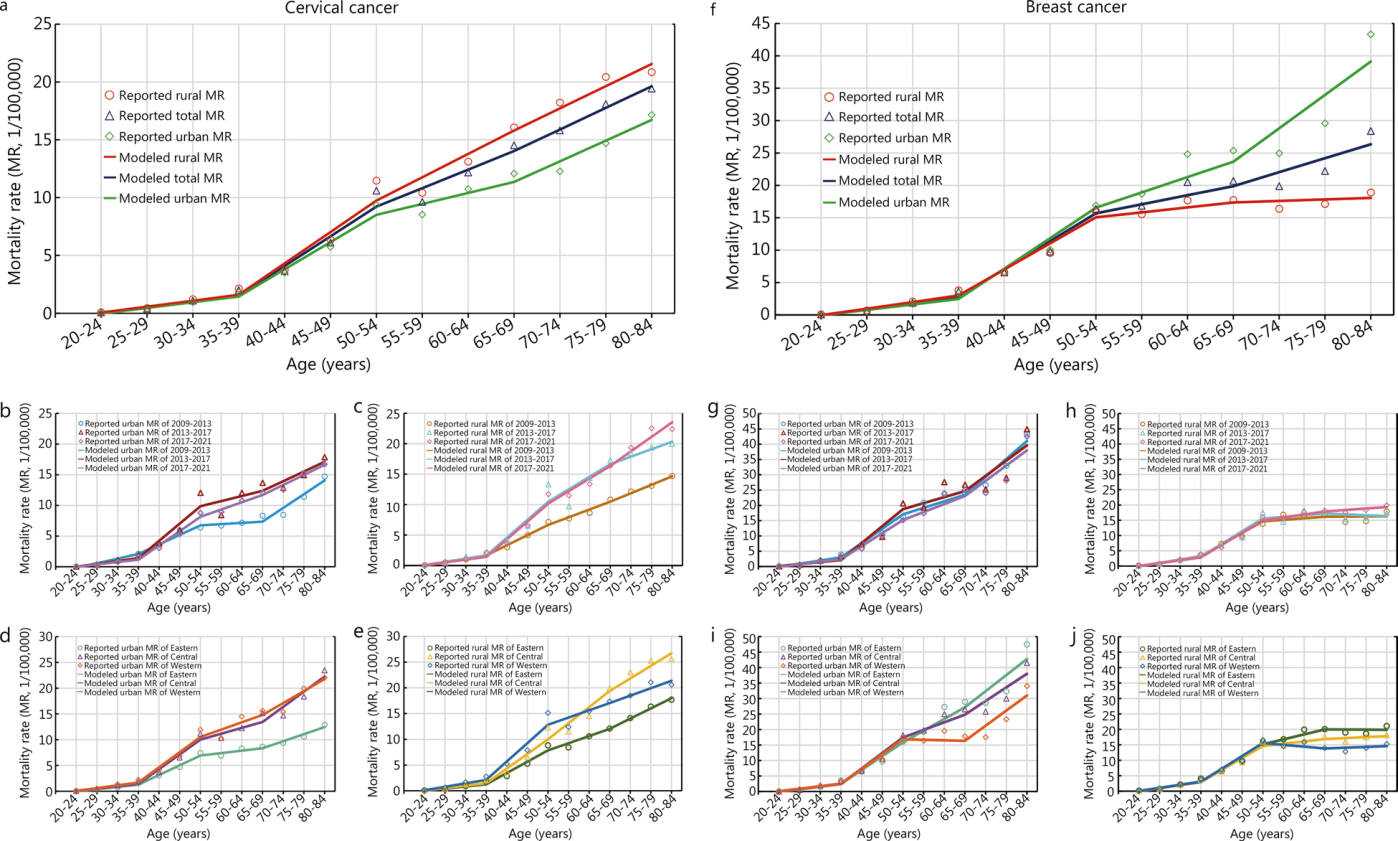
Mortality trends with age group for cervical cancer and breast cancer by residence, period, and region in China, 2009–2021. **a** cervical cancer mortality trend with age group by residence. **b** cervical cancer mortality trend with age group by period in urban areas. **c** cervical cancer mortality trend with age group by period in rural areas. **d** cervical cancer mortality trend with age group by region in urban areas. **e** cervical cancer mortality trend with age group by region in rural areas. **f** breast cancer mortality trend with age group by residence. **g** breast cancer mortality trend with age group by period in urban areas. **h** breast cancer mortality trend with age group by period in rural areas. **i** breast cancer mortality trend with age group by region in urban areas. **j** breast cancer mortality trend with age group by region in rural areas

Joinpoint regression identified four segments to evaluate mortality rate trends by age group, revealing differences in mortality acceleration patterns between urban and rural areas for the two cancers. The acceleration for the cervical cancer mortality rate segments were ranked as follows: segment 2, ages 35–39 to 50–54, with rates of 2.35 (urban) and 2.71 (rural), ranked 1st for both areas; segment 3, ages 50–54 to 65–69, with rates of 0.95 (urban) and 2.02 (rural), ranked 3rd in urban and 2nd in rural areas; segment 4, ages 65–69 to 80–84, with rates of 1.79 (urban) and 1.92 (rural), ranked 2nd in urban and 3rd in rural areas; and segment 1, ages 20–24 to 35–39, with rates of 0.49 (urban) and 0.51 (rural), ranked 4th in both areas. When stratified by period and region, the highest mortality acceleration was consistently found in segment 2, except for the urban 2009–2013 time period and in Central China (Table 3, Fig. 2a–e). Because 2017 is the Joinpoint in the mortality rate trends for the two cancers and is the intermediate time point after the system upgrade, it was chosen as the reference year for comparing the patterns in mortality acceleration with age for cervical and breast cancers against all cancers. Compared with the all-cancer mortality acceleration rate of 2017 (grey dashed line), the slopes of cervical cancer mortality were slightly higher in segment 1 and much higher in segment 2 (ages 35–39 to 50–54) for both areas (Additional file 1: Fig. S3a). For segments 3 and 4, among urban women, the acceleration increased more slowly than the all-cancers line across all periods. Among rural women, the mortality acceleration in the 2017–2021 time period was nearly parallel with the reference line (Additional file 1: Fig. S3a). Regionally, the slopes of cervical cancer mortality acceleration consistently showed slightly higher rates in segment 1 and much higher rates in segment 2 (Additional file 1: Fig. S3b). For segments 3 and 4, among urban women in Eastern China, the acceleration increased much more slowly than the all-cancers line. Among rural women in Central China, the mortality acceleration was nearly parallel to the reference line (Additional file 1: Fig. S3b).

**Table 3:** Mortality acceleration with age group for cervical cancer and breast cancer, period-specific and residence-specific in China, 2009–2021

Cancer type	Residence	Segment 1	Joinpoint 1 (years)	Segment 2	Joinpoint 2 (years)	Segment 3	Joinpoint 3 (years)	Segment 4
		Acceleration (SE, 1/100,000)		Acceleration (SE, 1/100,000)		Acceleration (SE, 1/100,000)		Acceleration (SE, 1/100,000)
Cervical cancer								
Year								
2009–2021	Urban	0.49 (0.07)^*^	35–39	2.35 (0.15)^*^	50–54	0.95 (0.15)^*^	65–69	1.79 (0.07)^*^
	Rural	0.51 (0.38)	35–39	2.71 (0.75)	50–54	2.02 (0.75)	65–69	1.92 (0.38)^*^
2009–2013	Urban	0.76 (0.11)^*^	35–39	1.55 (0.22)^*^	50–54	0.20 (0.22)	65–69	2.24 (0.11)^*^
	Rural	0.57 (0.14)	35–39	1.66 (0.28)^*^	50–54	1.27 (0.28)^*^	65–69	1.39 (0.14)^*^
2013–2017	Urban	0.47 (0.20)	35–39	2.82 (0.41)^*^	50–54	0.84 (0.41)	65–69	1.58 (0.20)^*^
	Rural	0.53 (0.15)	35–39	2.95 (0.29)^*^	50–54	2.04 (0.29)^*^	65–69	1.23 (0.15)^*^
2017–2021	Urban	0.39 (0.24)	35–39	2.33 (0.49)^*^	50–54	1.19 (0.49)	65–69	1.64 (0.24)^*^
	Rural	0.46 (0.69)	35–39	2.92 (1.37)	50–54	2.06 (1.37)	65–69	2.39 (0.69)
Region								
Eastern	Urban	0.45 (0.24)	35–39	1.89 (0.48)	50–54	0.46 (0.48)	65–69	1.38 (0.24)^*^
	Rural	0.42 (0.19)	35–39	2.19 (0.38)^*^	50–54	1.39 (0.38)	65–69	1.99 (0.19)^*^
Central	Urban	0.45 (0.30)	35–39	2.87 (0.60)^*^	50–54	1.14 (0.60)	65–69	2.95 (0.30)^*^
	Rural	0.45 (0.41)	35–39	2.84 (0.81)	50–54	3.13 (0.81)	65–69	2.44 (0.41)^*^
Western	Urban	0.54 (0.51)	35–39	2.94 (1.02)	50–54	1.42 (1.02)	65–69	2.33 (0.51)^*^
	Rural	0.67 (0.63)	35–39	3.56 (1.27)	50–54	1.41 (1.27)	65–69	1.44 (0.63)
Breast cancer								
Year								
2009–2021	Urban	0.84 (1.86)	35–39	4.70 (3.72)	50–54	2.37 (3.72)	65–69	5.16 (1.86)
	Rural	1.02 (0.27)	35–39	4.02 (0.54)^*^	50–54	0.77 (0.54)	65–69	0.23 (0.27)
2009–2013	Urban	1.03 (0.85)	35–39	4.69 (1.71)	50–54	2.14 (1.71)	65–69	5.86 (0.85)^*^
	Rural	1.12 (0.63)	35–39	3.81 (1.25)	50–54	0.51 (1.25)	65–69	0.04 (0.63)
2013–2017	Urban	0.68 (2.47)	35–39	5.55 (4.95)	50–54	1.96 (4.95)	65–69	4.99 (2.47)
	Rural	1.01 (0.30)	35–39	4.17 (0.60)^*^	50–54	0.55 (0.60)	65–69	− 0.27 (0.30)
2017–2021	Urban	0.84 (2.12)	35–39	4.28 (4.24)	50–54	2.62 (4.24)	65–69	4.98 (2.12)
	Rural	0.95 (0.22)	35–39	4.04 (0.44)	50–54	0.98 (0.44)	65–69	0.49 (0.22)
Region								
Eastern	Urban	0.88 (2.35)	35–39	4.46 (4.69)	50–54	3.78 (4.69)	65–69	5.19 (2.35)
	Rural	1.07 (0.61)	35–39	4.02 (1.23)	50–54	1.62 (1.23)	65–69	− 0.04 (0.61)
Central	Urban	0.81 (1.51)	35–39	5.00 (3.02)	50–54	2.46 (3.02)	65–69	4.40 (1.51)
	Rural	0.98 (0.21)^*^	35–39	3.89 (0.43)^*^	50–54	0.75 (0.43)	65–69	0.32 (0.21)
Western	Urban	0.82 (1.01)	35–39	4.78 (2.03)	50–54	− 0.17 (2.03)	65–69	4.88 (1.01)^*^
	Rural	0.98 (0.14)^*^	35–39	4.19 (0.27)^*^	50–54	− 0.59 (0.27)	65–69	0.25 (0.14)

^*^P < 0.05, the *Z* test was used to identify the significance of the slope (acceleration) obtained from the linear Joinpoint model from zero. *SE* standard error

The breast cancer mortality accelerations (per 100,000) by segment were as follows: segment 2, ages 35–39 to 50–54, with rates of 4.70 (urban) and 4.02 (rural), ranked 2nd in urban and 1st in rural areas; segment 1, ages 20–24 to 35–39, with rates of 0.84 (urban) and 1.02 (rural), ranked 4th in urban and 2nd in rural areas; segment 3, ages 50–54 to 65–69, with rates of 2.37 (urban) and 0.77 (rural), ranked 3rd for both areas; and segment 4, ages 65–69 to 80–84, with rates of 5.16 (urban) and 0.23 (rural), ranked 1st in urban and 4th in rural areas. Stratifying by period and region, the highest mortality acceleration for breast cancer was consistently observed in rural women in segment 2 (Table 3, Fig. 2f–j). For women aged 35–39 to 50–54 years, the mortality acceleration slope was steep and then diverged parallel to the reference population, being lower in rural senior-aged women, while for urban women it increased steeply compared to the reference line (Additional file 1: Fig. S4a). The mortality acceleration trends for breast cancer exhibited a different trajectory compared to cervical cancer; urban women in Western China exhibited slower mortality acceleration in segment 3, and then increased similarly to the rest of the regions, while rural women in segments 3 and 4 exhibited much lower acceleration than the reference line (Additional file 1: Fig. S4b). The major mortality peak for the two cancers was observed in the 35–54 age range.

## Discussion

This study investigates the temporal trends in age-specific mortality rates of cervical cancer and breast cancer from 2009 to 2021 in China, with a focus on women aged 35–64 years, 20–34 years, and 65–84 years. An increasing trend in cervical cancer mortality was observed in rural areas among women aged 35–64 years, contrasting with a stable trend in urban areas. This trend may be partially attributed to the implementation of the Program, which facilitates earlier diagnosis and enhances the accuracy of cause-of-death classification among rural women aged 35–64 years. Before the Program, significant urban–rural disparities existed in data quality and medical resources in China, including the underdevelopment of medical services in rural areas [30].

For cervical cancer screening, a limited number of rural medical facilities were capable of providing gynecological Pap smear testing or visual inspection with acetic acid or Lugol’s iodine (VIA/VILI) examinations. Additionally, diagnostic and therapeutic services such as colposcopy, cytology, and pathology were frequently unavailable in rural areas [31]. As a result, rural women experienced higher mortality rates, likely attributable to insufficient early diagnosis and treatment of precancerous lesions. In response to this challenge, the Program recommended improved services for cervical cancer prevention, including regular gynecological examination for initial screening, either cervical cytology or VIA/VILI examinations in areas lacking cytology, followed by appropriate colposcopy and pathology diagnoses. These improvements were implemented in 2009 [9], with further expansion of services occurring in 2015 [10].

High rates of cervical cancer-related deaths have been consistently recorded across all age groups in rural areas. Women in urban areas tend to avail themselves of cytological screening more frequently compared to their rural counterparts. Opportunistic screening for cervical intraepithelial neoplasia (CIN) by Pap smear testing is included in China's public healthcare system and has become standard practice at outpatient gynecology clinics located within city hospitals [8]. However, these services are predominantly accessible to urban residents who typically undergo employer-funded annual health check-ups that include cervical cytological screenings, thus highlighting the need for improved resource allocation and staffing for rural area-based cervical cancer screenings. Additionally, there has been an observable decline in the mortality rate due to cervical cancer among young urban women aged 20–34 years. This downward trend can be partly attributed to findings from a national study on sexually transmitted disease (STD) conducted between 2008 and 2016, which indicated reduced incidences of HPV-induced condyloma acuminatum among younger women, potentially indicative of declining oncogenic HPV as well [32]. Moreover, increasing awareness about HPV vaccines coupled with expanded efforts towards both early diagnosis through enhanced screenings and effective treatment options are likely contributing factors behind this positive development.

The mortality rate for breast cancer among urban women has shown a decreasing trend, while it has been increasing among rural women. Urban–rural differences in breast cancer mortality have been observed across all age groups except for the 20–24 and 40–44 age groups, with urban breast cancer mortality surpassing rural mortality. Factors such as prolonged menstrual periods, fewer births, later age at first birth, and limited breastfeeding in urban women may contribute to the higher incidence of breast cancer in urban areas compared to rural areas [33]. Urban women are more likely to adopt cosmopolitan lifestyles including higher consumption of energy-dense foods, increased alcohol intake, greater use of hormone replacement therapy and oral contraceptives, and higher exposure to exogenous estrogens and other environmental endocrine disruptors (EEDs), as well as higher rates of obesity and overweight—all associated with elevated breast cancer prevalence [2, 14, 34]. Recommended breast cancer screening procedures have evolved: initially in 2009, involving breast clinical examination and breast ultrasound examinations with expanded use of mammography [9]; then in 2015 using clinical breast examinations and ultrasound examinations first followed by mammography and/or histopathology assessments as needed [10]. With the implementation of health policies and healthy lifestyle initiatives, a promising decreasing trend in breast cancer mortality has been observed in the urban population; however, it remains unclear if the Program interventions were solely responsible for this improvement.

In the stratified analysis by period and region, consistent findings revealed higher cervical cancer mortality in rural women compared to urban women, whereas breast cancer mortality was higher in urban areas than in rural areas. The urban–rural disparities in mortality are increasing for cervical cancer and decreasing for breast cancer over time. The escalating urban–rural gap in cervical cancer mortality may be attributed to the decreasing mortality rate in the 35–49-year age group in urban populations and the implementation of cervical cancer screening in rural areas, leading to the discovery of more cases and more accurate attribution of deaths, particularly in the 55–59-year age group. The decreasing urban–rural disparity in breast cancer mortality may be related to the convergence of lifestyles between urban and rural residents regarding reproductive, hormonal, and other relevant breast cancer risk factors. As socioeconomic development and urbanization progress, differences in risk factors such as dietary patterns, lifestyle, and physical activity are becoming less pronounced across the country, resulting in a narrowing gap in breast cancer mortality rates between urban and rural women over time [35]. A significant disparity between urban and rural areas was found concerning cervical cancer mortality rates within economically developed Eastern China, while a smaller difference was observed in less-developed Western China. Conversely, a wide urban–rural gap was identified for breast cancer mortality rates in Central China, with similar differences observed between Eastern and Western China. Key provinces with significant mortality rates or disparities between urban and rural areas in cervical cancer from 2017 to 2021 include Liaoning, Hunan, Guizhou, Guangdong, Shanxi, and Chongqing. These findings indicate the necessity of adjusting the implementation of the Program in these regions. Similarly, for breast cancer, notable provinces encompass Liaoning, Heilongjiang, Guangxi, Shandong, Fujian, Henan, and Sichuan. Meanwhile, the most economically underdeveloped provinces, Tibet and Xinjiang in Western China, exhibited the most substantial improvement in reducing the disparity between urban and rural areas. These findings suggest that a tailored approach may be required for implementing the Program to address cervical cancer and breast cancer across different regions and provinces while optimizing scarce health resources.

An upward trend has been observed in cervical and breast cancer mortality rates across different age groups. Notably, there is a consistent rise in mortality within the age range of 35–54 years across urban and rural areas as well as various periods and regions, highlighting an urgent need for early screening initiatives. The Chinese government launched the Program in July 2009, offering free screening for rural women aged 35–59 years until 2011 [9], initially covering 10 million women. Subsequent modifications made in 2015 expanded the eligible age group to 35–64 years while expanding coverage to additional regions [10]. By 2022, the Program had been further extended to include all women, regardless of their residential status, aged 35–64 years [11]. However, two national surveys revealed suboptimal participation rates in cervical cancer screening, with estimates of only 25.7% for women aged 35–64 years in counties covered by the Program before 2014 and 31.4% in 2015 [12, 13]. Low participation and limited coverage may explain why cervical and breast cancer mortality is not decreasing more significantly. To address this, the Program planned to increase the screening rate to over 50% by 2025 among urban and rural women aged 35–64 years [11]. These results emphasize the importance of focusing on cervical cancer screening among younger age groups of women in both urban and rural areas, given the mortality surge in 35–54-year-olds.

Over time, there is a convergence of acceleration pattern disparities between urban and rural areas. Notably, within urban populations, changes in breast cancer mortality rates exhibit greater prominence among older women as opposed to their younger counterparts; conversely within rural populations such changes are less noticeable. Nevertheless, over successive periods, stability persists regarding the divergence of acceleration patterns between urban and rural locales. Specific provinces exhibiting elevated age-specific mortality rates have been earmarked for prioritized future screening efforts. Marked geographical discrepancies exist concerning mortality ratios for both types of cancers under consideration. Encouragingly, Tibet has narrowed its urban–rural gap in cervical cancer mortality while Xinjiang has mitigated its disparity in breast cancer mortalities, possibly attributable to socioeconomic advancements within these regions.

The main strength of this study lies in the comprehensive age-specific mortality data obtained from the DSP system, ensuring a representative sample of the national population. The use of stratified analysis methods maximizes information utilization to generate stable trends for each subgroup. Comparing modeled acceleration across different subgroups directly enhances result interpretation more concisely and visually. Despite high volatility in mortality rates within the 20–24, 25–29, and 30–34-year age groups due to low death counts, the results in the 20–34-year age group remained stable and consistent, bolstering robust conclusions. However, limitations include potential underestimation of mortality in certain young age groups due to underreporting. Additionally, attributing causation is precluded by the population-based study design. Conclusions regarding urban–rural differences are based on informed speculations due to a lack of specific socio-economic information, HPV vaccination rates, and cervical and breast cancer screening rates. Furthermore, model misspecification bias is not accounted for in model selection or reported 95% CIs.

## Conclusions

The present study has identified a notable upward trend in age-specific mortality for cervical and breast cancers in rural areas compared to urban areas. It also presents subnational urban–rural mortality ratio rankings. The primary increase in mortality was observed among women aged 35–54 years for both types of cancers. Despite significant government investment, national coverage rates for breast and cervical cancer screening remain low. Therefore, strategically prioritizing key age groups for urban (breast cancer) and rural (cervical cancer) screening in specific high-incidence provinces could optimize the utilization of available health resources. These findings underscore the importance of enhancing early screening for women aged 35–54 years old, 10 years earlier than the current screening age group, as well as regular medical examinations for Chinese seniors concerning both cancers.

### Supplementary Information

**Additional file 1**. National Diseases Surveillance Points (DSP) system. **Table S1** Number of disease surveillance points (DSPs) and underreporting rates of the DSP system in China, 2009–2021. **Table S2** Exploration analysis of temporal mortality trends for the two cancers among women aged 20–84 years in China, 2009–2021. **Table S3** Temporal trends in mortality rates by age group for cervical cancer and breast cancer in China, 2009–2021. **Table S4** Mortality rate ratios between urban and rural areas by period and region in China, 2009–2021. **Table S5** Age-specific cervical cancer mortality rate ratios between urban and rural areas by period and region in China, 2009–2021. **Table S6** Age-specific breast cancer mortality rate ratios between urban and rural areas by period and region in China, 2009–2021. **Fig. S1** Reported mortality rates for cervical cancer and breast cancer among women aged 20–84 years by age group in urban and rural areas in China, 2009–2021. **Fig. S2** Age-specific mortality rate ratios between urban and rural areas by period and region in China, 2009–2021. **Fig. S3** Mortality trends with age group for cervical cancer by residence, period, and region in China, 2009–2021. **Fig. S4** Mortality trends with age group for breast cancer by residence, period, and region in China, 2009–2021

## Data Availability

The datasets generated and/or analyzed during the current study are not publicly available but are available from the corresponding author with valid rationales for their use.

## Abbreviations

APC: Annual percent change
AAPC: Average annual percent change
ASIR: Age-standardized incidence rate
CDC: Center for Disease Control and Prevention
CI: Confidence interval
CIN: Cervical intraepithelial neoplasia
DSP: Diseases Surveillance Points
EED: Environmental endocrine disruptor
HPV: Human papillomavirus
ICD: International Classification of Diseases
IRB: Institutional review board
STD: Sexually transmitted disease
STI: Sexually transmittable infection
VIA/VILI: Virsual inspection with acetic acid or Lugol’s iodine
