# Supplementary material for: National age-specific mortality trends for cervical and breast cancers in urban–rural areas of China from 2009 to 2021: a population-based analysis
Source: Mil Med Res. 2024 Aug 13;11:55. doi: 10.1186/s40779-024-00561-4 (PMC11323448; doi:10.1186/s40779-024-00561-4)
Supplement: Supplementary file 1 — Additional file 1. National Diseases Surveillance Points (DSP) system. Table S1 Number of disease surveillance points (DSPs) and underreporting rates of the DSP system in China, 2009–2021. Table S2 Exploration analysis of temporal mortality trends for the two cancers among women aged 20–84 years in China, 2009–2021. Table S3 Temporal trends in mortality rates by age group for cervical cancer and breast cancer in China, 2009–2021. Table S4 Mortality rate ratios between urban and rural areas by period and region in China, 2009–2021. Table S5 Age-specific cervical cancer mortality rate ratios between urban and rural areas by period and region in China, 2009–2021. Table S6 Age-specific breast cancer mortality rate ratios between urban and rural areas by period and region in China, 2009–2021. Fig. S1 Reported mortality rates for cervical cancer and breast cancer among women aged 20–84 years by age group in urban and rural areas in China, 2009–2021. Fig. S2 Age-specific mortality rate ratios between urban and rural areas by period and region in China, 2009–2021. Fig. S3 Mortality trends with age group for cervical cancer by residence, period, and region in China, 2009–2021. Fig. S4 Mortality trends with age group for breast cancer by residence, period, and region in China, 2009–2021 [file 40779_2024_561_MOESM1_ESM.pdf]

National Diseases Surveillance Points (DSP) system

History of the national DSP system

The development of China's sample-based disease surveillance point (DSP) system began in 1978 with a pilot study at two surveillance points in Beijing. By 1989, the system expanded to 71 surveillance points across 29 provinces, formally establishing the national DSP system [1]. In 1990, supported by the World Bank and the Ministry of Health (now the National Health Commission), the Chinese Academy of Preventive Medicine [Chinese Center for Disease Control and Prevention (now China CDC)] selected representative points in 31 provinces to form a new DSP system. This new system used multistage stratified whole-group random sampling, expanding to 145 disease surveillance points, and routinely collecting information on births, population, and deaths, covering a total of 10 million people under surveillance (about 1% of China's total population) [2]. In 2003, the DSP system was further expanded to 161 surveillance sites across 31 provinces, covering more than 77 million people, or about 6% of the national population. By 2013, the National Health and Family Planning Commission (now the National Health Commission) integrated and expanded the cause of death reporting systems. This integration increased the number of surveillance points to 605, covering 300 million persons or roughly 24% of the national population [3].

DSP selection after 2013

Firstly, the National Health and Family Planning Commission established that the surveillance population should be not less than 5 million in provinces with a population greater than 10 million and that were economically well developed. For provinces with less than 10 million, the surveillance population had to be at least 20% of the total population. These criteria determined the number of required surveillance points per province. Secondly, all districts and counties in each province were stratified into eight strata based on urbanization degree, population size, and crude mortality rate (total deaths per 1000 population per year). Representative districts (urban population) and counties (rural population) in each stratum were selected as candidate surveillance points according to the required number of points. Data from the 2010 census were then used to determine the representativeness of these candidate surveillance points within each province. The final selection of surveillance points involved an iterative process to ensure the representativity of the provincial population. After multiple rounds of evaluation and adjustment, a total of 605 surveillance points was chosen across China, with the number of points per province ranging from 7 to 36. Three of the former DSP systems with 161 points were excluded due to poor data quality and limited support and capacity from local governments [3].

DSP reporting process

The DSP System registers all deaths occurring within each surveillance point, including those of residents, immigrants from other regions, Hong Kong, Macao, and Taiwan compatriots, and foreign citizens. All types and levels of medical and health institutions are responsible for reporting cause of death information. Only medical and health personnel with medical practitioner qualifications are permitted to fill out the Medical Certificate of Cause of Death. All deaths in the national cause of death surveillance system are reported online through the China CDC’s cause of death registration reporting information system. The China CDC audits the data reported by provinces and verifies and corrects the reporting bias or mistakes [2].

Quality control and improvement of the DSP system

Two methods are employed for controlling data quality within the DSP system. The first is an internal procedural check system, which evaluates the timeliness of death registration, the completeness of entries in the registration form, and the accuracy of data entry. Errors detected from these checks are corrected through re-enquiry, enhancing the usability of the datasets. The second method involves evaluating the complete datasets using statistical measures. The completeness and accuracy of population enumeration in the DSP system are assessed using the standard United Nations Age Sex Accuracy Index [1].

References

1. Yang G, Hu J, Rao KQ, Ma J, Rao C, Lopez AD. Mortality registration and surveillance in China: history, current situation and challenges. Popul Health Metr. 2005;3(1):3.
2. NCNCD, China CDC. Data sets of death cause surveillance in disease surveillance points system. Available online: https://ncncd.chinacdc.cn/xzzq_1/202101/t20210111_223706.htm (accessed on 1 June 2024).
3. Liu S, Wu X, Lopez AD, Wang L, Cai Y, Page A, et al. An integrated national mortality surveillance system for death registration and mortality surveillance, China. Bull World Health Organ. 2016;94(1):46-57.

Table S1 Number of disease surveillance points (DSPs) and underreporting rates of the disease surveillance point (DSP) system in China, 2009 – 2021

**Region**	**Number of DSP**		**Underreporting rate (%)**		
	**2009 − 2012**	**2013 − 2021**	**2009 − 2011**	**2012 − 2014**	**2015 − 2021**
Eastern	54	209	16.5	12.2	6.1
Central	50	192	12.2	13.5	10.9
Western	57	204	25.4	30.3	12.8

Table S2 Exploration analysis of temporal mortality trends for the two cancers among women aged 20 – 84 years in China, 2009 – 2021

**Cancer type**	**Mortality rate (1/100,000)**				**AAPC [%(95% CI)]**	**APC [%(95% CI)]**		
	**2009**	**2013**	**2017**	**2021**	**2009 − 2021**	**2009 − 2013**	**2013 − 2017**	**2017 − 2021**
Cervical cancer								
Reported	4.51	4.36	7.06	6.25	3.0 (1.2 − 4.8) *	3.1 (-0.1 to 6.5)	12.0 (6.5 − 17.8) *	-5.5 (-8.4 to -2.4) *
Modeled	4.31	4.88	7.67	6.12				
Breast cancer								
Reported	9.95	8.78	10.11	10.00	0 (-1.0 to 1.0)	-2.6 (-4.4 to -0.8) *	2.8 (-0.2 to 5.9)	-0.2 (-2.1 to 1.6)
Modeled	10.03	9.03	10.09	9.99				

*$P < 0.05$, the Z test was used to identify the significance of the *APC*, *AAPC* obtained from the log-linear Joinpoint model from zero. *AAPC* average annual percent change, *APC* annual percent change, *CI* confidence interval

Table S3 Temporal trends in mortality rates by age group for cervical cancer and breast cancer in China, 2009 – 2021

Age group (years)	Mortality rate (1/100,000, 95% CI)								AAPC (%, 95% CI)		Pooled mortality rate in 2009 – 2021 (1/100,000, 95% CI)		
	2009		2013		2017		2021						
	Urban	Rural	Urban	Rural	Urban	Rural	Urban	Rural	Urban	Rural	Urban	Rural	Ratio
Cervical cancer													
20 − 24	0.11 (0 − 0.57)	0.06 (0 − 0.28)	0.06 (0.02 − 0.16)	0.04 (0.01 − 0.11)	0.03 (0 − 0.11)	0.06 (0.02 − 0.14)	0.03 (0 − 0.16)	0.12 (0.04 − 0.27)	27.1 (-2.5 to 65.7)	23.6 (-4.7 to 60.3)	0.05 (0.03 − 0.07)	0.09 (0.07 − 0.12)	0.55 (0.36 − 0.85) *
25 − 29	0.41 (0.13 − 0.98)	0.30 (0.09 − 0.71)	0.25 (0.14 − 0.43)	0.37 (0.24 − 0.54)	0.28 (0.16 − 0.44)	0.49 (0.34 − 0.69)	0.23 (0.12 − 0.40)	0.40 (0.24 − 0.61)	-7.1 (-11.1 to -2.9) *	-4.4 (-9.4 to 1.0)	0.32 (0.27 − 0.37)	0.45 (0.40 − 0.50)	0.71 (0.59 − 0.85) *
30 − 34	1.71 (1.00 − 2.73)	0.86 (0.48 − 1.43)	0.86 (0.61 − 1.18)	0.93 (0.70 − 1.21)	0.99 (0.73 − 1.30)	1.41 (1.12 − 1.76)	0.47 (0.32 − 0.65)	0.68 (0.51 − 0.90)	-5.7 (-9.5 to -1.8) *	-1.5 (-7.6 to 5.1)	0.95 (0.87 − 1.03)	1.22 (1.14 − 1.31)	0.77 (0.69 − 0.87) *
35 − 39	2.23 (1.51 − 3.19)	1.80 (1.29 − 2.45)	2.06 (1.70 − 2.48)	1.95 (1.65 − 2.29)	1.80 (1.47 − 2.18)	2.37 (2.02 − 2.76)	1.32 (1.04 − 1.64)	2.19 (1.84 − 2.60)	-3.9 (-5.5 to -2.3) *	0.8 (-2.0 to 3.7)	1.82 (1.71 − 1.94)	2.14 (2.03 − 2.25)	0.85 (0.79 − 0.92) *
40 − 44	4.11 (3.09 − 5.37)	2.90 (2.24 − 3.70)	3.85 (3.35 − 4.40)	2.97 (2.62 − 3.35)	3.06 (2.63 − 3.54)	4.03 (3.61 − 4.48)	2.24 (1.86 − 2.67)	3.04 (2.62 − 3.51)	-2.2 (-5.3 to 1.0)	1.6 (-2.6 to 6.0)	3.50 (3.34 − 3.65)	3.75 (3.62 − 3.89)	0.93 (0.88 − 0.99) *
45 − 49	7.38 (5.87 − 9.16)	5.44 (4.33 − 6.75)	5.19 (4.63 − 5.79)	4.56 (4.14 − 5.00)	5.83 (5.26 − 6.44)	7.45 (6.92 − 8.01)	4.23 (3.73 − 4.78)	5.44 (4.93 − 5.98)	-3.3 (-5.2 to -1.4) *	1.6 (-1.1 to 4.3)	5.74 (5.55 − 5.93)	6.37 (6.21 − 6.54)	0.90 (0.86 − 0.94) *
50 − 54	7.05 (5.64 − 8.70)	5.24 (4.17 − 6.50)	6.81 (5.99 − 7.71)	8.22 (7.46 − 9.04)	14.29 (13.12 − 15.54)	17.04 (15.99 − 18.14)	6.37 (5.77 − 7.01)	8.93 (8.29 − 9.61)	-2.1 (-11.6 to 8.3)	4.1 (-0.6 to 9.1)	9.38 (9.10 − 9.66)	11.47 (11.21 − 11.73)	0.82 (0.79 − 0.85) *
55 − 59	7.59 (5.98 − 9.50)	8.03 (6.59 − 9.69)	6.71 (5.95 − 7.53)	7.48 (6.83 − 8.17)	8.30 (7.48 − 9.18)	10.06 (9.30 − 10.86)	8.27 (7.54 − 9.06)	12.04 (11.22 − 12.90)	2.9 (0.7 - 5.2) *	4.5 (2.9 - 6.0) *	8.53 (8.27 − 8.80)	10.42 (10.17 − 10.68)	0.82 (0.79 − 0.85) *
60 − 64	6.61 (4.76 − 8.94)	8.68 (6.88 − 10.82)	7.33 (6.38 − 8.37)	8.72 (7.91 − 9.59)	14.05 (12.76 − 15.43)	17.17 (16.05 − 18.34)	9.08 (8.17 − 10.07)	10.99 (10.09 − 11.94)	2.7 (-2.5 to 8.3)	1.4 (-5.1 to 8.4)	10.74 (10.39 − 11.10)	13.12 (12.81 − 13.44)	0.82 (0.79 − 0.85) *

65 − 69	9.39	12.17	9.04	10.50	15.68	19.28	9.47	14.57	-0.1	2.7	12.08	16.08	0.75
	(6.90 − 12.49)	(9.67 − 15.11)	(7.75 − 10.48)	(9.40 − 11.69)	(14.10 − 17.39)	(17.92 − 20.72)	(8.52 − 10.50)	(13.57 − 15.61)	(-7.8 to 8.1)	(-3.8 to 9.7)	(11.65 − 12.51)	(15.69 − 16.48)	(0.72 − 0.78) *
70 − 74	7.12	12.74	8.82	12.40	12.95	20.30	11.02	18.03	4.7	2.8	12.27	18.22	0.67
	(4.85 − 10.08)	(9.99 − 16.01)	(7.43 − 10.40)	(11.03 − 13.88)	(11.40 − 14.66)	(18.67 − 22.03)	(9.78 − 12.39)	(16.68 − 19.46)	(0.3 - 9.2) *	(-4.6 to 10.8)	(11.79 − 12.77)	(17.73 − 18.72)	(0.64 − 0.71) *
75 − 79	14.49	14.15	11.37	12.83	15.80	20.16	14.26	22.71	0.3	6.1	14.70	20.44	0.72
	(10.51 − 19.49)	(10.73 − 18.32)	(9.61 − 13.35)	(11.30 − 14.50)	(13.92 − 17.87)	(18.39 − 22.05)	(12.55 − 16.14)	(20.81 − 24.73)	(-7.3 to 8.6)	(3.7 - 8.6) *	(14.09 − 15.32)	(19.85 − 21.05)	(0.68 − 0.76) *
80 − 84	19.76	18.64	12.36	14.12	18.03	20.84	14.80	22.24	-0.3	1.0	17.16	20.86	0.82
	(13.57 − 27.80)	(13.55 − 25.01)	(10 − 15.10)	(12.09 − 16.40)	(15.40 − 20.97)	(18.66 − 23.21)	(12.76 − 17.07)	(20.08 − 24.57)	(-3.2 to 2.6)	(-4.5 to 6.9)	(16.33 − 18.02)	(20.13 − 21.61)	(0.77 − 0.87) *
Breast cancer													
20 − 24	0.12	0.16	0.16	0.14	0.08	0.07	0.06	0.05	-10.1	-7.2	0.08	0.10	0.74
	(0 − 0.58)	(0.04 − 0.43)	(0.07 − 0.29)	(0.07 − 0.24)	(0.03 − 0.18)	(0.02 − 0.15)	(0.01 − 0.20)	(0.01 − 0.16)	(-16.5 to -3.2) *	(-15.2 to 1.6)	(0.06 − 0.10)	(0.08 − 0.13)	(0.51 − 1.08)
25 − 29	0.91	0.62	0.50	0.75	0.59	0.72	0.46	0.83	-5.0	2.7	0.50	0.70	0.72
	(0.44 − 1.65)	(0.30 − 1.15)	(0.33 − 0.72)	(0.56 − 0.99)	(0.41 − 0.81)	(0.54 − 0.95)	(0.30 − 0.68)	(0.59 − 1.12)	(-10.5 to 0.8)	(-0.2 to 5.8)	(0.45 − 0.56)	(0.64 − 0.76)	(0.62 − 0.83) *
30 − 34	2.49	1.56	1.67	1.86	2.18	2.52	1.38	1.90	-4.2	0.6	1.68	2.07	0.81
	(1.62 − 3.68)	(1.02 − 2.27)	(1.31 − 2.09)	(1.52 − 2.24)	(1.79 − 2.63)	(2.12 − 2.97)	(1.13 − 1.67)	(1.60 − 2.24)	(-7.9 to -0.4) *	(-2.6 to 4.0)	(1.58 − 1.80)	(1.96 − 2.19)	(0.75 − 0.88) *
35 − 39	4.96	3.98	3.63	3.43	3.24	4.17	3.36	3.75	-3.7	-0.1	3.45	3.81	0.90
	(3.84 − 6.30)	(3.19 − 4.90)	(3.14 − 4.18)	(3.02 − 3.87)	(2.79 − 3.74)	(3.71 − 4.67)	(2.90 − 3.86)	(3.28 − 4.27)	(-6.5 to -0.7) *	(-1.3 to 1.1)	(3.30 − 3.60)	(3.67 − 3.96)	(0.85 − 0.96) *
40 − 44	7.93	7.69	7.02	7.19	6.48	6.33	5.05	5.85	-3.4	-2.2	6.62	6.58	1.01
	(6.48 − 9.61)	(6.58 − 8.93)	(6.34 − 7.76)	(6.64 − 7.76)	(5.85 − 7.17)	(5.81 − 6.89)	(4.48 − 5.68)	(5.27 − 6.49)	(-5.0 to -1.7) *	(-3.5 to -0.9) *	(6.41 − 6.83)	(6.40 − 6.76)	(0.96 − 1.05)
45 − 49	14.29	11.28	9.49	8.64	10.39	9.70	8.79	9.28	-4.1	-1.8	10.07	9.59	1.05
	(12.16 − 16.70)	(9.65 − 13.10)	(8.74 − 10.29)	(8.07 − 9.24)	(9.62 − 11.20)	(9.09 − 10.34)	(8.06 − 9.56)	(8.62 − 9.99)	(-6.7 to -1.5) *	(-3.2 to -0.3) *	(9.82 − 10.33)	(9.39 − 9.79)	(1.02 − 1.09) *
50 − 54	17.36	13.60	16.10	14.29	22.72	20.74	11.10	13.17	-3.0	-0.9	16.89	16.01	1.05
	(15.11 − 19.85)	(11.84 − 15.55)	(14.83 − 17.45)	(13.28 − 15.36)	(21.24 − 24.28)	(19.58 − 21.95)	(10.31 − 11.94)	(12.39 − 13.98)	(-7.2 to 1.5)	(-4.7 to 3.1)	(16.52 − 17.27)	(15.71 − 16.33)	(1.02 − 1.09) *

55 − 59	23.69 (20.77 − 26.90)	18.83 (16.59 − 21.29)	20.66 (19.32 − 22.08)	15.22 (14.29 − 16.20)	17.28 (16.09 − 18.54)	14.11 (13.20 − 15.05)	16.18 (15.15 − 17.26)	17.56 (16.57 − 18.60)	-2.4 (-3.6 to -1.2) *	-1.2 (-3.0 to 0.7)	18.68 (18.29 − 19.08)	15.55 (15.24 − 15.85)	1.20 (1.17 − 1.24) *
60 − 64	20.54 (17.15 − 24.39)	17.61 (14.99 − 20.54)	24.26 (22.53 − 26.10)	15.74 (14.65 − 16.90)	31.59 (29.64 − 33.63)	20.16 (18.95 − 21.43)	18.66 (17.34 − 20.06)	15.72 (14.65 − 16.85)	-1.2 (-4.6 to 2.3)	0 (-1.7 to 1.7)	24.84 (24.30 − 25.38)	17.70 (17.33 − 18.06)	1.40 (1.36 − 1.45) *
65 − 69	26.67 (22.33 − 31.60)	16.38 (13.46 − 19.75)	23.83 (21.70 − 26.10)	16.66 (15.27 − 18.14)	28.94 (26.78 − 31.23)	20.29 (18.90 − 21.77)	23.37 (21.85 − 24.96)	16.01 (14.96 − 17.10)	-1.3 (-4.2 to 1.7)	-0.6 (-3.5 to 2.5)	25.38 (24.76 − 26.00)	17.75 (17.34 − 18.16)	1.43 (1.38 − 1.48) *
70 − 74	31.53 (26.51 − 37.22)	14.61 (11.65 − 18.09)	24.93 (22.54 − 27.49)	14.72 (13.23 − 16.33)	23.72 (21.59 − 25.99)	16.40 (14.94 − 17.96)	24.62 (22.73 − 26.61)	18.07 (16.72 − 19.51)	-1.7 (-2.9 to -0.4) *	2.2 (0.6 - 3.9) *	24.97 (24.28 − 25.68)	16.41 (15.94 − 16.88)	1.52 (1.46 − 1.58) *
75 − 79	39.65 (32.85 − 47.44)	18.19 (14.28 − 22.85)	28.90 (26.06 − 31.97)	13.50 (11.93 − 15.21)	25.67 (23.25 − 28.27)	16.20 (14.62 − 17.90)	29.02 (26.56 − 31.65)	21.48 (19.64 − 23.45)	-2.7 (-4.2 to -1.2) *	2.1 (0.2 - 4.0) *	29.60 (28.74 − 30.48)	17.13 (16.59 − 17.69)	1.73 (1.65 − 1.80) *
80 − 84	40.36 (31.26 − 51.28)	23.51 (17.74 − 30.55)	41.75 (37.31 − 46.56)	15.50 (13.36 − 17.88)	47.69 (43.35 − 52.35)	18.69 (16.62 − 20.93)	40.24 (36.83 − 43.88)	19.63 (17.61 − 21.83)	-0.5 (-1.6 to 0.6)	-0.9 (-3.7 to 2.0)	43.32 (42.00 − 44.68)	18.92 (18.22 − 19.63)	2.29 (2.18 − 2.40) *

*$P < 0.05$, the *Z* test was used to identify the significance of the *AAPC* obtained from the log-linear Joinpoint model from zero, the chi-square test was used to identify the significance of the mortality rate ratios between urban and rural areas (reference) obtained from the negative binomial regression model from one. *AAPC* average annual percent change, *CI* confidence interval

Table S4 Mortality rate ratios between urban and rural areas by period and region in China, 2009 – 2021

Cancer type	Mortality rate (1/100,000, 95% CI) by age (years)											
	20 − 84			20 − 34			35 − 64			65 − 84		
	Urban	Rural	Ratio	Urban	Rural	Ratio	Urban	Rural	Ratio	Urban	Rural	Ratio
Cervical cancer												
Year												
2009 − 2013	4.17 (4.04 − 4.30)	4.53 (4.42 − 4.65)	0.92 (0.88 − 0.96) *	0.45 (0.38 − 0.53)	0.46 (0.40 − 0.53)	0.98 (0.79 − 1.22)	5.05 (4.87 − 5.23)	5.06 (4.91 − 5.22)	1.00 (0.95 − 1.05)	9.90 (9.33 − 10.49)	12.22 (11.70 − 12.76)	0.81 (0.75 − 0.87) *
2013 − 2017	5.47 (5.38 − 5.56)	7.01 (6.93 − 7.10)	0.78 (0.76 − 0.80) *	0.44 (0.39 − 0.48)	0.58 (0.54 − 0.63)	0.75 (0.66 − 0.85) *	6.58 (6.45 − 6.71)	7.62 (7.50 − 7.73)	0.86 (0.84 − 0.99) *	14.33 (13.91 − 14.77)	18.44 (18.06 − 18.83)	0.78 (0.75 − 0.81) *
2017 − 2021	5.37 (5.28 − 5.45)	7.73 (7.64 − 7.82)	0.69 (0.68 − 0.71) *	0.39 (0.35 − 0.44)	0.53 (0.49 − 0.58)	0.74 (0.64 − 0.84) *	6.02 (5.90 − 6.14)	7.85 (7.73 − 7.97)	0.77 (0.75 − 0.79) *	13.50 (13.14 − 13.86)	19.18 (18.83 − 19.53)	0.70 (0.68 − 0.73) *
Region												
Eastern	4.08 (4.01 − 4.16)	5.73 (5.64 − 5.81)	0.71 (0.70 − 0.73) *	0.32 (0.29 − 0.36)	0.41 (0.37 − 0.45)	0.80 (0.68 − 0.93) *	5.01 (4.90 − 5.12)	6.03 (5.91 − 6.14)	0.83 (0.81 − 0.86) *	9.90 (9.58 − 10.24)	14.40 (14.05 − 14.76)	0.69 (0.66 − 0.72) *
Central	6.31 (6.18 − 6.45)	7.88 (7.77 − 7.99)	0.80 (0.78 − 0.82) *	0.50 (0.44 − 0.58)	0.57 (0.51 − 0.63)	0.89 (0.75 − 1.05)	7.02 (6.83 − 7.22)	7.84 (7.69 − 7.98)	0.90 (0.87 − 0.93) *	16.73 (16.12 − 17.37)	22.62 (22.13 − 23.12)	0.74 (0.71 − 0.77) *
Western	6.74 (6.60 − 6.88)	8.00 (7.87 − 8.13)	0.84 (0.82 − 0.86) *	0.61 (0.54 − 0.69)	0.78 (0.70 − 0.86)	0.79 (0.67 − 0.93) *	7.40 (7.21 − 7.59)	8.90 (8.72 − 9.08)	0.83 (0.80 − 0.86) *	17.15 (16.55 − 17.77)	18.84 (18.31 − 19.38)	0.91 (0.87 − 0.95) *
Breast cancer												
Year												

2009 − 2013	10.75	8.12	1.32	0.79	0.79	1.00	12.46	10.36	1.20	29.28	15.79	1.85
	(10.54 − 10.95)	(7.97 − 8.27)	(1.29− 1.36) [*]	(0.69 − 0.89)	(0.71 − 0.88)	(0.85− 1.18)	(12.17 − 12.75)	(10.14 − 10.58)	(1.17− 1.24) [*]	(28.30 − 30.29)	(15.20 − 16.40)	(1.76 − 1.95) [*]
2013 − 2017	10.75	8.74	1.23	0.77	0.89	0.86	12.74	10.84	1.18	29.34	16.71	1.76
	(10.63 − 10.88)	(8.65 − 8.84)	(1.21− 1.25) [*]	(0.71 − 0.83)	(0.83 − 0.94)	(0.78− 0.95) [*]	(12.56 − 12.93)	(10.70 − 10.98)	(1.15− 1.20) [*]	(28.74 − 29.96)	(16.35 − 17.07)	(1.70 − 1.81) [*]
2017 − 2021	10.63	9.56	1.11	0.71	0.95	0.75	11.68	11.11	1.05	27.81	18.28	1.52
	(10.51 − 10.75)	(9.46 − 9.66)	(1.09− 1.13) [*]	(0.65 − 0.77)	(0.89 − 1.01)	(0.67− 0.83) [*]	(11.51 − 11.85)	(10.97 − 11.26)	(1.03− 1.07) [*]	(27.30 − 28.34)	(17.94 − 18.62)	(1.48 − 1.56) [*]
Region												
Eastern	11.15	9.82	1.14	0.66	0.86	0.76	12.53	11.60	1.08	32.42	19.61	1.65
	(11.02 − 11.27)	(9.71 − 9.93)	(1.12− 1.15) [*]	(0.60 − 0.71)	(0.80 − 0.93)	(0.68− 0.85) [*]	(12.36 − 12.72)	(11.44 − 11.76)	(1.06− 1.10) [*]	(31.83 − 33.02)	(19.20 − 20.03)	(1.61 − 1.70) [*]
Central	11.14	8.77	1.27	0.81	0.89	0.92	12.57	10.51	1.20	28.92	17.26	1.68
	(10.96 − 11.33)	(8.65 − 8.88)	(1.24− 1.30) [*]	(0.73 − 0.91)	(0.82 − 0.96)	(0.80− 1.05)	(12.31 − 12.83)	(10.35 − 10.68)	(1.17− 1.23) [*]	(28.11 − 29.75)	(16.83 − 17.69)	(1.61 − 1.74) [*]
Western	9.42	8.19	1.15	0.87	0.98	0.89	11.09	10.36	1.07	20.70	13.77	1.50
	(9.25 − 9.58)	(8.06 − 8.32)	(1.12− 1.18) [*]	(0.78 − 0.97)	(0.89 − 1.07)	(0.77− 1.02)	(10.86 − 11.33)	(10.17 − 10.56)	(1.04− 1.10) [*]	(20.03 − 21.37)	(13.32 − 14.24)	(1.43 − 1.57) [*]

[*] $P < 0.05$, the chi-square test was used to identify the significance of the mortality rate ratios between urban and rural areas (reference) obtained from the negative binomial regression model from one. *CI* confidence interval

Table S5 Age-specific cervical cancer mortality rate ratios between urban and rural areas by period and region in China, 2009 – 2021

Age group (years)	Mortality rate (1/100,000, 95% CI) by period									Mortality rate (1/100,000, 95% CI) by region								
	2009 − 2013			2013 − 2017			2017 − 2021			Eastern			Central			Western		
	Urban	Rural	Ratio	Urban	Rural	Ratio	Urban	Rural	Ratio	Urban	Rural	Ratio	Urban	Rural	Ratio	Urban	Rural	Ratio
20 − 24	0.04 (0.01 – 0.10)	0.04 (0.02 – 0.08)	1.02 (0.33 – 3.22)	0.04 (0.02 – 0.07)	0.09 (0.06 – 0.12)	0.49 (0.26 – 0.89) *	0.06 (0.04 – 0.10)	0.10 (0.07 – 0.15)	0.59 (0.32 – 1.08)	0.03 (0.02 – 0.06)	0.05 (0.03 – 0.08)	0.72 (0.32 – 1.62)	0.05 (0.02 – 0.10)	0.12 (0.08 – 0.17)	0.42 (0.18 – 0.96) *	0.09 (0.05 – 0.15)	0.13 (0.08 – 0.19)	0.69 (0.35 – 1.37)
25 − 29	0.38 (0.27 – 0.52)	0.52 (0.40 – 0.66)	0.74 (0.50 – 1.09)	0.34 (0.27 – 0.41)	0.46 (0.39 – 0.54)	0.73 (0.57 – 0.94) *	0.26 (0.20 – 0.32)	0.40 (0.34 – 0.48)	0.64 (0.48 – 0.84) *	0.21 (0.16 – 0.27)	0.33 (0.26 – 0.40)	0.65 (0.47 – 0.89) *	0.41 (0.31 – 0.54)	0.47 (0.38 – 0.57)	0.87 (0.63 – 1.22)	0.49 (0.38 – 0.63)	0.64 (0.52 – 0.77)	0.77 (0.56 – 1.05)
30 − 34	1.04 (0.84 – 1.27)	1.01 (0.84 – 1.20)	1.03 (0.79 – 1.35)	1.10 (0.97 – 1.25)	1.48 (1.34 – 1.63)	0.75 (0.64 – 0.87) *	0.79 (0.70 – 0.90)	1.06 (0.96 – 1.18)	0.74 (0.63 – 0.88) *	0.72 (0.63 – 0.83)	0.91 (0.80 – 1.04)	0.79 (0.66 – 0.95) *	1.11 (0.93 – 1.32)	1.24 (1.10 – 1.40)	0.90 (0.73 – 1.10)	1.34 (1.14 – 1.56)	1.70 (1.50 – 1.91)	0.79 (0.65 – 0.96) *
35 − 39	2.13 (1.87 – 2.41)	2.01 (1.80 – 2.25)	1.05 (0.89 – 1.25)	1.95 (1.79 – 2.12)	2.19 (2.04 – 2.34)	0.89 (0.80 – 0.99) *	1.62 (1.48 – 1.78)	2.16 (2.00 – 2.32)	0.75 (0.67 – 0.85) *	1.56 (1.42 – 1.72)	1.77 (1.61 – 1.93)	0.89 (0.78 – 1.01)	1.92 (1.70 – 2.17)	2.03 (1.86 – 2.22)	0.95 (0.81 – 1.10)	2.21 (1.98 – 2.46)	2.78 (2.55 – 3.02)	0.80 (0.70 – 0.91) *
40 − 44	3.59 (3.25 – 3.96)	3.00 (2.75 – 3.26)	1.20 (1.05 – 1.36) *	3.86 (3.64 – 4.10)	4.10 (3.91 – 4.30)	0.94 (0.87 – 1.01)	3.06 (2.86 – 3.27)	3.57 (3.38 – 3.77)	0.86 (0.79 – 0.93) *	3.05 (2.85 – 3.27)	2.86 (2.67 – 3.05)	1.07 (0.97 – 1.17)	3.68 (3.37 – 4.01)	3.64 (3.42 – 3.87)	1.01 (0.91 – 1.12)	4.10 (3.78 – 4.43)	5.23 (4.92 – 5.54)	0.78 (0.71 – 0.87) *
45 − 49	5.96 (5.53 – 6.42)	4.99 (4.65 – 5.34)	1.20 (1.08 – 1.32) *	5.97 (5.71 – 6.25)	6.53 (6.31 – 6.76)	0.91 (0.86 –0.97) *	5.33 (5.08 – 5.59)	6.55 (6.31 – 6.79)	0.81 (0.77 – 0.86) *	4.67 (4.43 – 4.92)	5.27 (5.03 – 5.51)	0.89 (0.83 – 0.95) *	6.53 (6.14 – 6.94)	6.51 (6.24 – 6.80)	1.00 (0.93 – 1.08)	6.86 (6.47 – 7.27)	7.90 (7.53 – 8.27)	0.87 (0.81 – 0.94) *
50 − 54	6.42 (5.90 – 6.98)	7.17 (6.68 – 7.69)	0.90 (0.80 – 1.00) *	12.06 (11.57 – 12.57)	13.33 (12.90 – 13.76)	0.91 (0.86 – 0.95) *	8.79 (8.44 – 9.16)	11.72 (11.38 – 12.08)	0.75 (0.71 – 0.79) *	7.41 (7.07 – 7.76)	8.84 (8.50 – 9.20)	0.84 (0.79 – 0.89) *	11.17 (10.56 – 11.81)	12.31 (11.85 – 12.78)	0.91 (0.85 – 0.97) *	11.94 (11.29 – 12.61)	15.15 (14.53 – 15.80)	0.79 (0.74 – 0.84) *

55 – 59	6.71 (6.18 – 7.27)	7.71 (7.22 – 8.22)	0.87 (0.79 – 0.96) *	8.43 (8.05 – 8.82)	9.73 (9.39 – 10.07)	0.87 (0.82 – 0.92) *	8.93 (8.55 – 9.32)	11.48 (11.11 – 11.86)	0.78 (0.74 – 0.82) *	6.91 (6.58 – 7.25)	8.43 (8.09 – 8.79)	0.82 (0.77 – 0.87) *	10.33 (9.73 – 10.95)	11.52 (11.07 – 11.98)	0.90 (0.84 – 0.96) *	10.25 (9.67 – 10.86)	12.40 (11.84 – 12.98)	0.83 (0.77 – 0.89) *
60 – 64	7.16 (6.49 – 7.88)	8.63 (8.03 – 9.27)	0.83 (0.74 – 0.93) *	12.07 (11.53 – 12.64)	14.38 (13.92 – 14.86)	0.84 (0.79 – 0.89) *	10.79 (10.32 – 11.28)	13.37 (12.94 – 13.81)	0.81 (0.76 – 0.85) *	8.25 (7.82 – 8.69)	10.61 (10.18 – 11.06)	0.78 (0.73 – 0.83) *	12.25 (11.49 – 13.04)	14.54 (13.99 – 15.10)	0.84 (0.78 – 0.91) *	14.49 (13.67 – 15.33)	15.49 (14.79 – 16.22)	0.94 (0.87 – 1.01)
65 – 69	8.33 (7.47 – 9.28)	10.84 (10.03 – 11.69)	0.77 (0.67 – 0.88) *	13.70 (13.00 – 14.43)	17.26 (16.65 – 17.88)	0.79 (0.75 – 0.85) *	12.10 (11.56 – 12.66)	16.34 (15.83 – 16.86)	0.74 (0.70 – 0.78) *	8.63 (8.13 – 9.16)	12.11 (11.59 – 12.65)	0.71 (0.66 – 0.77) *	15.17 (14.21 – 16.18)	19.91 (19.18 – 20.67)	0.76 (0.71 – 0.82) *	15.61 (14.69 – 16.56)	17.37 (16.56 – 18.21)	0.90 (0.83 – 0.97) *
70 – 74	8.47 (7.52 – 9.50)	12.15 (11.19 – 13.17)	0.70 (0.61 – 0.80) *	12.89 (12.15 – 13.66)	18.44 (17.71 – 19.19)	0.70 (0.65 – 0.75) *	12.58 (11.93 – 13.26)	19.36 (18.69 – 20.05)	0.65 (0.61 – 0.69) *	9.36 (8.75 – 9.99)	14.14 (13.47 – 14.83)	0.66 (0.61 – 0.72) *	14.68 (13.61 – 15.80)	23.05 (22.10 – 24.03)	0.64 (0.59 – 0.69) *	15.38 (14.34 – 16.48)	18.51 (17.54 – 19.53)	0.83 (0.76 – 0.91) *
75 – 79	11.39 (10.13 – 12.76)	13.07 (11.95 – 14.27)	0.87 (0.75 – 1.01)	14.94 (14.06 – 15.86)	19.35 (18.54 – 20.20)	0.77 (0.72 – 0.83) *	15.23 (14.40 – 16.10)	22.54 (21.68 – 23.43)	0.68 (0.63 – 0.72) *	10.57 (9.86 – 11.31)	16.36 (15.56 – 17.20)	0.65 (0.59 – 0.70) *	18.36 (16.98 – 19.81)	25.31 (24.17 – 26.50)	0.73 (0.66 – 0.79) *	19.94 (18.52 – 21.45)	21.09 (19.85 – 22.39)	0.95 (0.86 – 1.04)
80 – 84	14.75 (12.86 – 16.83)	14.68 (13.15 – 16.34)	1.00 (0.85 – 1.19)	17.88 (16.63 – 19.21)	19.97 (18.95 – 21.03)	0.90 (0.82 – 0.98) *	16.73 (15.65 – 17.86)	22.38 (21.36 – 23.43)	0.75 (0.69 – 0.81) *	12.92 (11.97 – 13.92)	17.71 (16.73 – 18.73)	0.73 (0.66 – 0.80) *	23.47 (21.41 – 25.68)	25.62 (24.20 – 27.10)	0.92 (0.82 – 1.02)	22.04 (20.04 – 24.19)	20.61 (19.04 – 22.27)	1.07 (0.95 – 1.21)

*$P < 0.05$, the chi-square test was used to identify the significance of the mortality rate ratios between urban and rural areas (reference) obtained from the negative binomial regression model from one. *CI* confidence interval

Table S6 Age-specific breast cancer mortality rate ratios between urban and rural areas by period and region in China, 2009 – 2021

Age	**Mortality rate (1/100,000, 95% CI) by period**									**Mortality rate (1/100,000, 95% CI) by region**								
**group**	**2009 − 2013**			**2013 − 2017**			**2017 − 2021**			**Eastern**			**Central**			**Western**		
**(years)**	**Urban**	**Rural**	**Ratio**	**Urban**	**Rural**	**Ratio**	**Urban**	**Rural**	**Ratio**	**Urban**	**Rural**	**Ratio**	**Urban**	**Rural**	**Ratio**	**Urban**	**Rural**	**Ratio**
20 − 24	0.14 (0.08 − 0.23)	0.15 (0.10 − 0.22)	0.93 (0.50 − 1.74)	0.09 (0.06 − 0.13)	0.10 (0.07 − 0.13)	0.96 (0.60 − 1.52)	0.05 (0.03 − 0.08)	0.09 (0.06 − 0.13)	0.55 (0.28 − 1.09)	0.08 (0.05 − 0.12)	0.08 (0.05 − 0.12)	1.01 (0.57 − 1.80)	0.05 (0.02 − 0.10)	0.11 (0.07 − 0.16)	0.45 (0.20 − 1.04)	0.10 (0.05 − 0.17)	0.14 (0.09 − 0.20)	0.74 (0.39 − 1.43)
25 − 29	0.57 (0.44 − 0.73)	0.64 (0.51 − 0.79)	0.89 (0.64 − 1.23)	0.56 (0.48 − 0.66)	0.73 (0.65 − 0.83)	0.77 (0.63 − 0.93) *	0.44 (0.37 − 0.52)	0.70 (0.61 − 0.80)	0.63 (0.50 − 0.78) *	0.38 (0.32 − 0.46)	0.60 (0.51 − 0.69)	0.64 (0.51 − 0.81) *	0.54 (0.42 − 0.69)	0.73 (0.62 − 0.85)	0.74 (0.56 − 0.98) *	0.75 (0.61 − 0.92)	0.82 (0.69 − 0.98)	0.91 (0.71 − 1.19)
30 − 34	1.86 (1.59 − 2.16)	1.89 (1.66 − 2.16)	0.98 (0.80 − 1.19)	1.95 (1.77 − 2.13)	2.27 (2.10 − 2.45)	0.86 (0.76 − 0.97) *	1.51 (1.37 − 1.65)	2.01 (1.86 − 2.16)	0.75 (0.67 − 0.84) *	1.50 (1.37 − 1.64)	2.06 (1.89 − 2.24)	0.73 (0.64 − 0.83) *	1.96 (1.72 − 2.22)	2.04 (1.85 − 2.24)	0.96 (0.82 − 1.12)	1.86 (1.63 − 2.13)	2.15 (1.93 − 2.39)	0.87 (0.73 − 1.03)
35 − 39	3.92 (3.56 − 4.30)	3.62 (3.33 − 3.93)	1.08 (0.96 − 1.23)	3.36 (3.15 − 3.58)	3.89 (3.69 − 4.10)	0.86 (0.79 − 0.94) *	3.34 (3.13 − 3.56)	3.81 (3.60 − 4.03)	0.88 (0.81 − 0.95) *	3.57 (3.35 − 3.80)	4.03 (3.79 − 4.27)	0.89 (0.81 − 0.97) *	3.39 (3.08 − 3.71)	3.70 (3.46 − 3.95)	0.92 (0.82 − 1.03)	3.28 (2.99 − 3.58)	3.67 (3.41 − 3.95)	0.89 (0.80 − 1.00)
40 − 44	7.25 (6.76 − 7.76)	7.30 (6.91 − 7.71)	0.99 (0.91 − 1.08)	7.22 (6.91 − 7.54)	6.83 (6.58 − 7.08)	1.06 (1.00 − 1.12)	5.80 (5.52 − 6.09)	6.03 (5.78 − 6.28)	0.96 (0.90 − 1.03)	6.38 (6.08 − 6.69)	6.50 (6.22 − 6.79)	0.98 (0.92 − 1.05)	6.73 (6.31 − 7.17)	6.39 (6.10 − 6.69)	1.05 (0.97 − 1.14)	6.92 (6.51 − 7.35)	6.94 (6.59 − 7.30)	1.00 (0.92 − 1.08)
45 − 49	10.92 (10.33 − 11.54)	9.80 (9.33 − 10.29)	1.11 (1.04 − 1.20) *	9.72 (9.38 − 10.07)	9.37 (9.10 − 9.65)	1.04 (0.99 − 1.09)	10.07 (9.72 − 10.43)	9.56 (9.28 − 9.85)	1.05 (1.01 − 1.10) *	9.70 (9.34 − 10.06)	9.70 (9.38 − 10.03)	1.00 (0.95 − 1.05)	10.49 (9.99 − 11.00)	9.31 (8.98 − 9.65)	1.13 (1.06 − 1.20) *	10.34 (9.86 − 10.83)	9.80 (9.40 − 10.22)	1.05 (0.99 − 1.12)

50 − 54	16.22 (15.38 − 17.09)	13.85 (13.16 − 14.57)	1.17 (1.09 − 1.26)*	20.64 (19.99 − 21.30)	17.51 (17.02 − 18.00)	1.18 (1.13 − 1.23)*	15.18 (14.71 − 15.66)	16.00 (15.60 − 16.42)	0.95 (0.91 − 0.99)*	16.11 (15.61 − 16.63)	15.75 (15.29 − 16.22)	1.02 (0.98 − 1.07)	18.17 (17.39 − 18.98)	16.02 (15.50 − 16.56)	1.13 (1.07 − 1.20)*	17.31 (16.54 − 18.12)	16.50 (15.85 − 17.17)	1.05 (0.99 − 1.11)
55 − 59	20.90 (19.96 − 21.88)	16.83 (16.11 − 17.57)	1.24 (1.17 − 1.32)*	19.27 (18.69 − 19.86)	14.54 (14.13 − 14.96)	1.33 (1.27 − 1.38)*	17.41 (16.89 − 17.95)	15.72 (15.29 − 16.16)	1.11 (1.06 − 1.15)*	19.25 (18.70 − 19.81)	16.76 (16.28 − 17.26)	1.15 (1.10 − 1.20)*	19.77 (18.95 − 20.63)	14.71 (14.20 − 15.22)	1.34 (1.27 − 1.42)*	16.41 (15.67 − 17.18)	14.59 (13.98 − 15.21)	1.13 (1.06 − 1.20)*
60 − 64	23.78 (22.55 − 25.06)	16.50 (15.66 − 17.37)	1.44 (1.34 – 1.55)*	27.59 (26.76 − 28.44)	18.28 (17.76 − 18.82)	1.51 (1.45 − 1.57)*	23.93 (23.22 − 24.65)	17.65 (17.15 − 18.16)	1.36 (1.30 − 1.41)*	27.30 (26.52 − 28.09)	19.90 (19.30 − 20.51)	1.37 (1.32 − 1.43)*	24.98 (23.90 − 26.10)	16.32 (15.73 − 16.92)	1.53 (1.45 − 1.62)*	19.56 (18.61 − 20.54)	15.83 (15.11 − 16.56)	1.24 (1.16 − 1.32)*
65 − 69	23.82 (22.34 − 25.38)	16.71 (15.70 − 17.76)	1.43 (1.31 − 1.56)*	26.78 (25.80 − 27.79)	17.87 (17.25 − 18.50)	1.50 (1.42 − 1.58)*	25.17 (24.38 − 25.97)	18.19 (17.66 − 18.74)	1.38 (1.32 − 1.44)*	28.97 (28.04 − 29.92)	20.06 (19.39 − 20.76)	1.44 (1.38 − 1.51)*	26.56 (25.28 − 27.88)	17.68 (16.99 − 18.39)	1.50 (1.41 − 1.60)*	17.81 (16.83 − 18.83)	13.94 (13.22 − 14.70)	1.28 (1.18 − 1.38)*
70 − 74	26.65 (24.94 − 28.44)	14.49 (13.44 − 15.60)	1.84 (1.67 − 2.03)*	25.20 (24.16 − 26.28)	15.46 (14.79 − 16.15)	1.63 (1.53 − 1.73)*	24.05 (23.15 − 24.98)	17.51 (16.87 − 18.17)	1.37 (1.30 − 1.45)*	28.63 (27.57 − 29.72)	18.95 (18.18 − 19.75)	1.51 (1.43 − 1.60)*	25.82 (24.40 − 27.29)	16.00 (15.21 − 16.82)	1.61 (1.50 − 1.74)*	17.55 (16.43 − 18.72)	12.81 (12.00 − 13.66)	1.37 (1.25 − 1.50)*
75 − 79	33.00 (30.83 − 35.27)	14.71 (13.52 − 15.98)	2.24 (2.02 − 2.49)*	29.01 (27.78 − 30.29)	15.86 (15.12 − 16.62)	1.83 (1.72 − 1.95)*	28.14 (27.01 − 29.32)	18.33 (17.55 − 19.13)	1.54 (1.45 − 1.63)*	32.33 (31.08 − 33.62)	18.58 (17.72 − 19.47)	1.74 (1.64 − 1.85)*	30.01 (28.24 − 31.85)	17.49 (16.55 − 18.48)	1.72 (1.58 − 1.86)*	23.34 (21.79 − 24.96)	13.94 (12.94 − 15.01)	1.67 (1.51 − 1.85)*
80 − 84	43.44 (40.16 − 46.92)	17.90 (16.21 − 19.73)	2.43 (2.14 − 2.75)*	44.90 (42.89 − 46.98)	17.41 (16.46 − 18.40)	2.58 (2.40 − 2.77)*	42.59 (40.87 − 44.37)	19.98 (19.02 − 20.97)	2.13 (2.00 − 2.27)*	47.50 (45.67 − 49.38)	21.06 (20.00 − 22.17)	2.26 (2.11 − 2.40)*	41.53 (38.77 − 44.43)	18.19 (17.00 − 19.44)	2.28 (2.08 − 2.51)*	34.07 (31.57 − 36.72)	15.23 (13.89 − 16.67)	2.24 (1.99 − 2.51)*

*$P < 0.05$, the chi-square test was used to identify the significance of the mortality rate ratios between urban and rural areas (reference) obtained from the negative binomial regression model from one. *CI* confidence interval

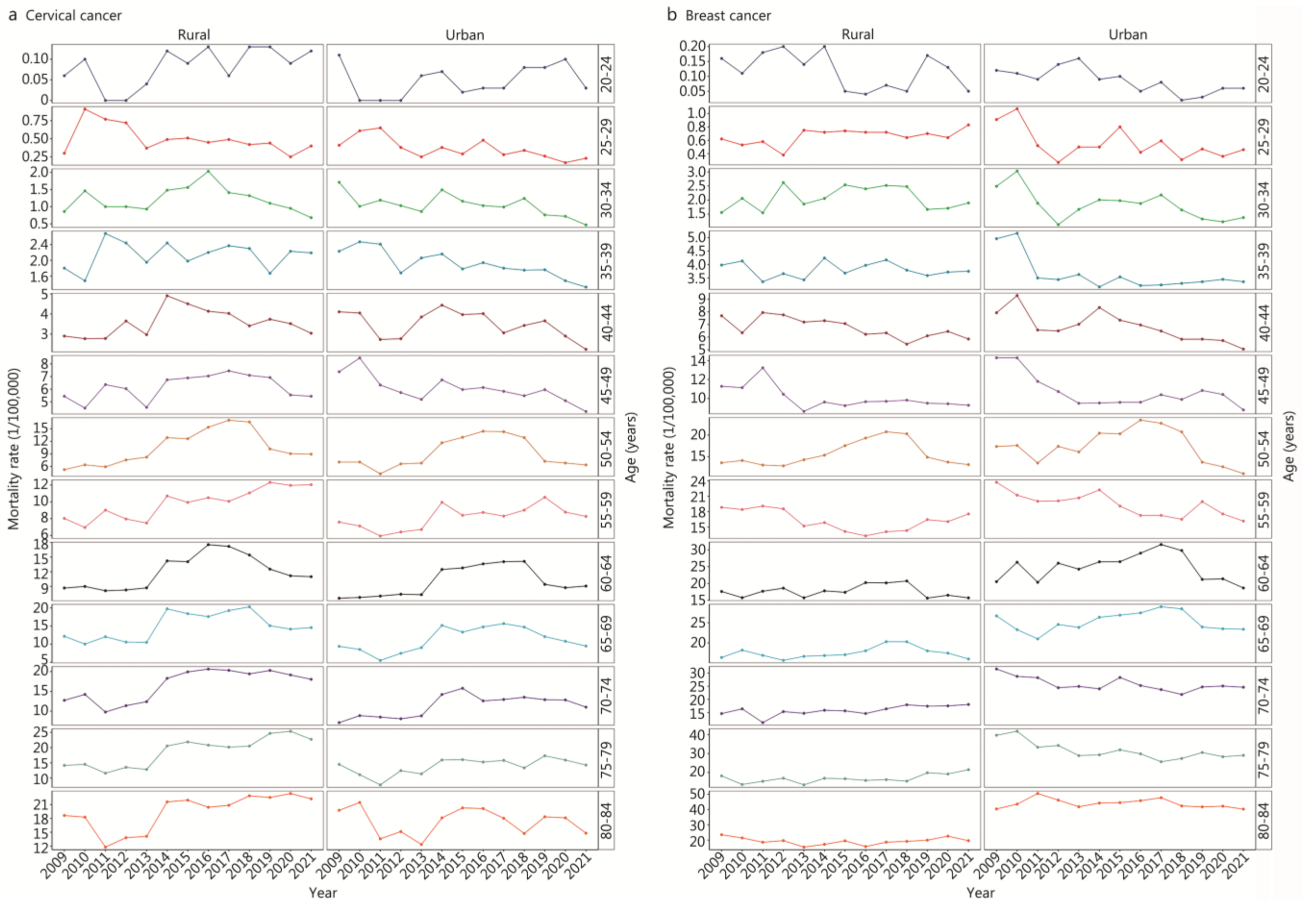

Fig. S1 Reported mortality rates for cervical cancer and breast cancer among women aged 20 – 84 years by age group and in urban and rural areas in China, 2009 – 2021. **a** Cervical cancer. **b** Breast cancer

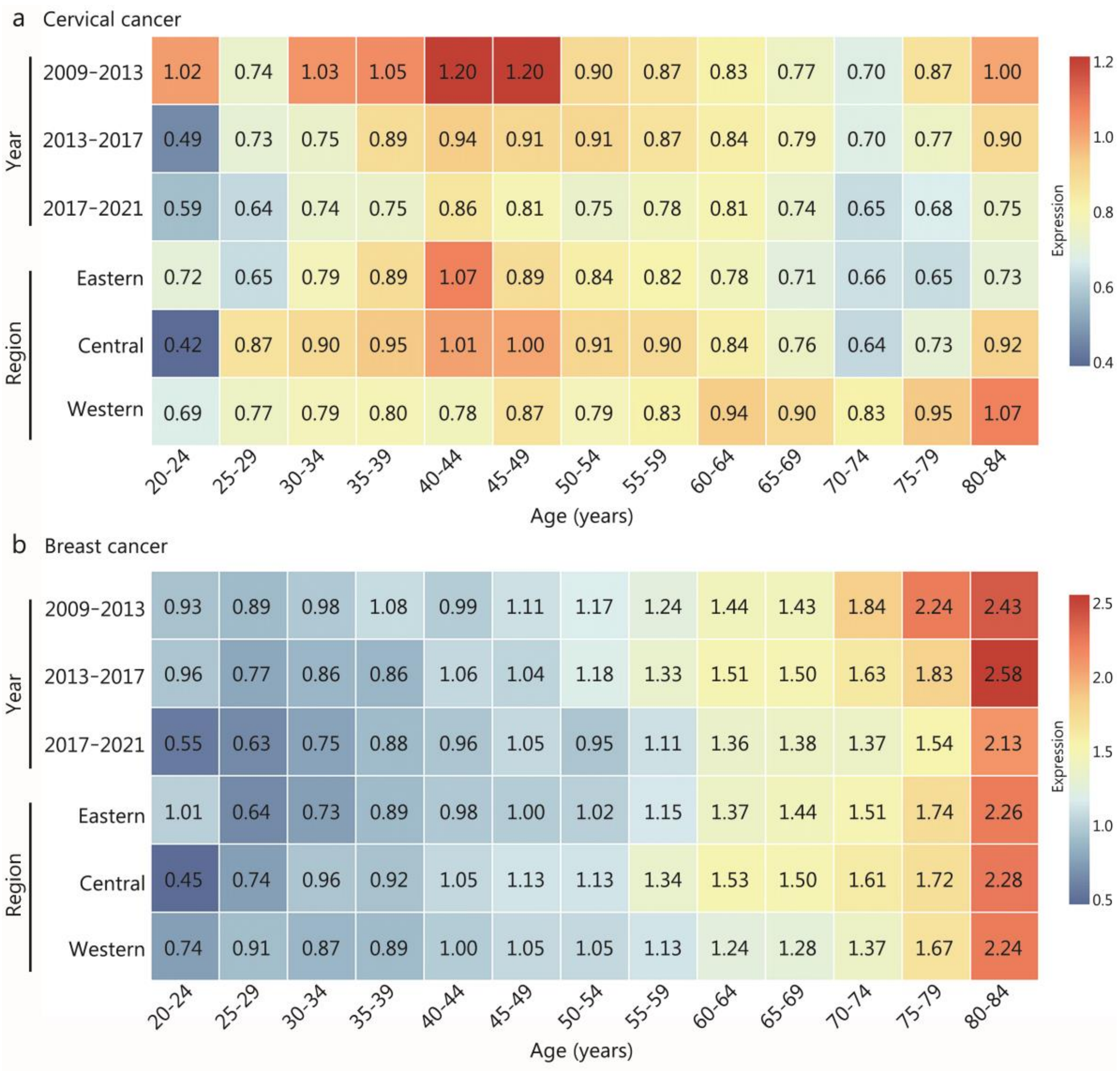

Fig. S2 Age-specific mortality rate ratios between urban and rural areas by period and region in China, 2009 – 2021. **a** Cervical cancer. **b** Breast cancer

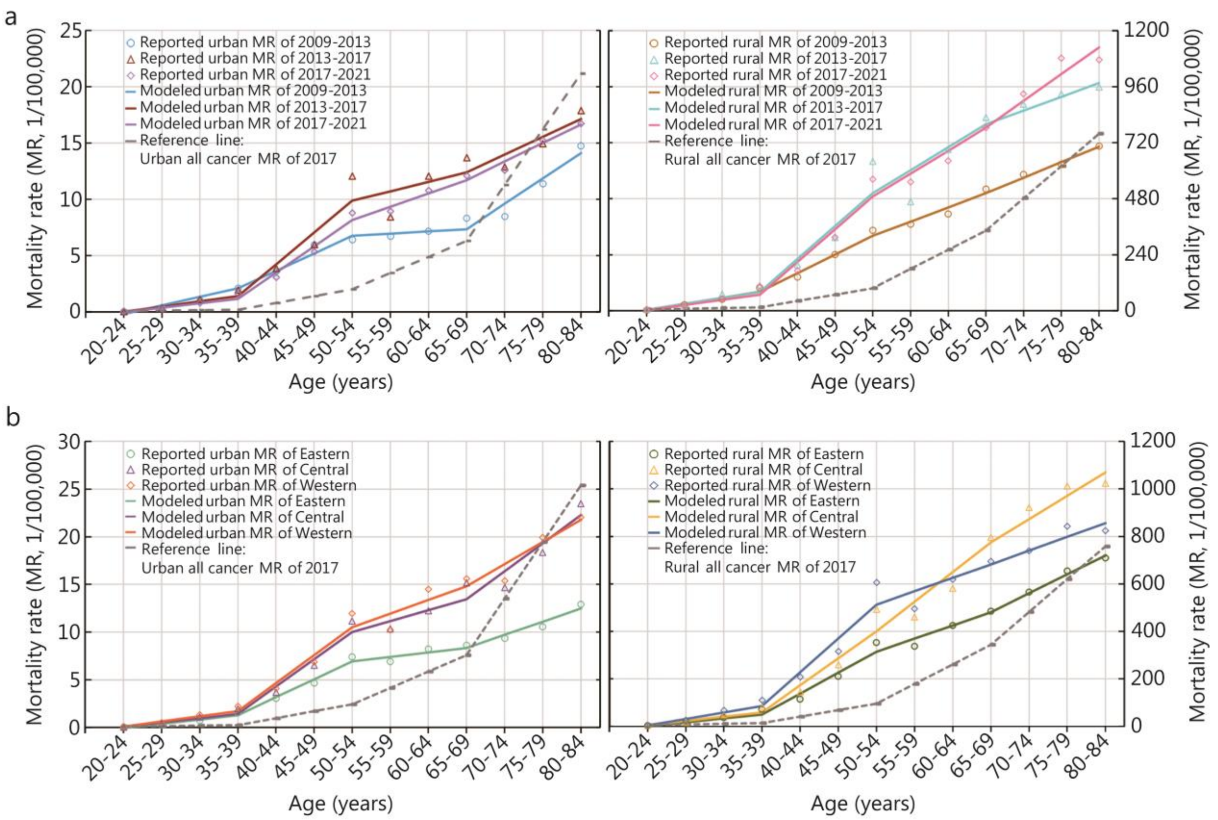

Fig. S3 Mortality trends with age group for cervical cancer by residence, period, and region in China, 2009 – 2021. **a** Mortality trend with age group by residence and period. **b** Mortality trend with age group by residence and region. The right Y-axis corresponds to the all-cancer mortality rate for 2017. MR mortality rate

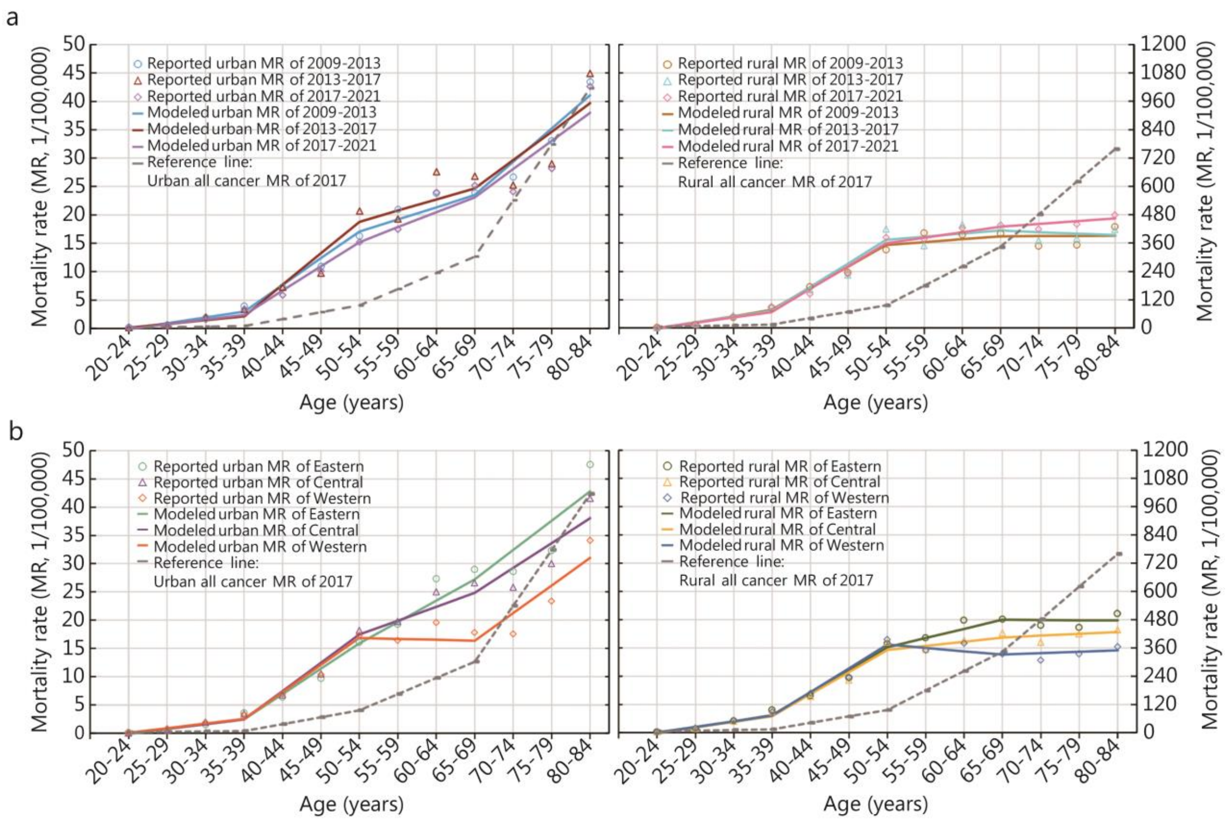

Fig. S4 Mortality trends with age group for breast cancer by residence, period, and region in China, 2009 – 2021. **a** Mortality trend with age group by residence and period. **b** Mortality trend with age group by residence and region. The right Y-axis corresponds to the all-cancer mortality rate for 2017. MR mortality rate
